# m6A demethylase FTO attenuates cardiac dysfunction by regulating glucose uptake and glycolysis in mice with pressure overload-induced heart failure

**DOI:** 10.1038/s41392-021-00699-w

**Published:** 2021-11-02

**Authors:** Beijian Zhang, Hao Jiang, Jian Wu, Yun Cai, Zhen Dong, Yongchao Zhao, Qinfeng Hu, Kai Hu, Aijun Sun, Junbo Ge

**Affiliations:** 1 Department of Cardiology, Zhongshan Hospital, Fudan University, Shanghai, China; 2 Shanghai Institute of Cardiovascular Diseases, Shanghai, China; 3 Key Laboratory of Viral Heart Diseases, National Health Commission, Shanghai, China; 4 Key Laboratory of Viral Heart Diseases, Chinese Academy of Medical Sciences, Shanghai, China; 5 Institutes of Biomedical Sciences, Fudan University, Shanghai, China

**Keywords:** Cardiovascular diseases, Cardiology

**Dear Editor**,

*N*^6^-methyladenosine (m6A) is the most common post-transcriptional mammalian mRNA modification involved in multiple biological processes and diseases.^1^ However, its role in cardiac energy metabolism changes during heart failure (HF) remains elusive. Here we explored the role of m6A demethylase fat mass and obesity-associated protein (FTO) in glucose metabolism during pressure overload-induced HF.

Methylated RNA immunoprecipitation sequencing (MeRIP-seq) was performed to map transcriptome-wide m6A modifications in transverse aortic constriction (TAC) and sham hearts. The number of identified m6A peaks was higher in the TAC than in the sham hearts (Fig. 1a). Consistent with previous reports, the m6A peaks were enriched in 3’-untranslated regions in both the TAC and sham hearts (Fig. 1b).^2^ The m6A peaks were characterized by the canonical RGAAR (R = A or G) motif (Fig. 1c). Gene ontology analysis revealed that the m6A changes after TAC were not randomly distributed throughout the genome but were instead specifically increased in certain mRNA classes, particularly in those related to metabolism (Fig. 1d). Subsequent Kyoto Encyclopedia of Genes and Genomes (KEGG) analysis revealed that glycolysis was significantly enriched, including five glycolysis-related genes such as aldolase B (*Aldob*), phosphoglycerate mutase 2 (*Pgam2*), phosphoglucomutase 2 (*Pgm2*), triosephosphate isomerase (*Tpi1*), and dihydrolipoyl dehydrogenase (*Dld*) (Fig. 1e). The mRNAs with altered m6A in the TAC-induced failing hearts are shown in Supplementary Table S1.

**Fig. 1:**
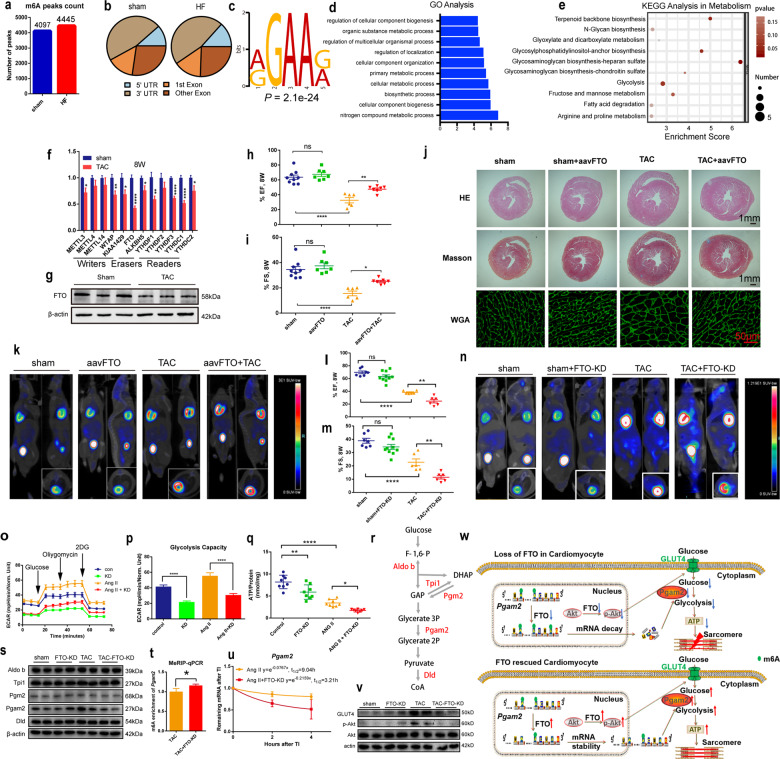
m6A demethylase FTO attenuates cardiac dysfunction by regulating glucose uptake and glycolysis in mice with pressure overload-induced heart failure. **a** Total m6A peaks identified by MeRIP-seq in sham and TAC mice. **b** Distribution of m6A peaks throughout mRNA lengths. **c** Sequence logo representing the consensus motif identified by Discriminative Regular Expression Motif Elicitation (DREME). **d** Top 10 enriched GO terms from MeRIP-seq data. **e** KEGG analysis of altered m6A methylation in metabolic mRNAs. **f** Quantification of m6A methyltransferase, demethylase, and methyl-specific binding protein mRNA levels in left ventricles of mice at 8 weeks (W) post-TAC (*n* = 6/group). **g** Immunoblot of FTO protein levels at 8 weeks post-TAC (*n* = 6/group). **h**, **i** Echocardiographic ejection fraction (EF) and fractional shortening (FS) measurements indicating left ventricle function at 8 weeks post-TAC (*n* = 6–9/group). **j** Representative images of hematoxylin and eosin (HE), Masson’s trichrome, and wheat germ agglutinin (WGA) staining. **k** Representative cardiac ^18^F-FDG uptake imaging by micro-PET/CT in FTO-overexpressed mice. **l**, **m** EF and FS at 8 weeks post-TAC after FTO knockdown (*n* = 6–10/group). **n** Representative cardiac ^18^F-FDG uptake imaging in mice with FTO knockdown. **o** Extracellular acidification rates (ECARs) of isolated cardiomyocytes using the Seahorse XF Analyzer. **p** Glycolytic function (*n* = 19–24/group). **q** ATP production (*n* = 8/group). **r** Glycolysis diagram with genes affected by m6A methylation (red). **s** Representative immunoblots of ALDOB, TPI1, PGM2, PGAM2, and DLD (*n* = 4/group). **t** Quantification of m6A enrichment of *Pgam2* mRNA detected by MeRIP-qPCR (*n* = 3/group). **u** Lifetime of *Pgam2* mRNA in primary cardiomyocytes by monitoring transcript abundance after transcription inhibition (TI) at different time points (*n* = 3/group). **v** Representative immunoblots of GLUT4 and p-AKT (*n* = 4/group). **w** Model of FTO regulation of glucose uptake and glycolysis and its potential translational value in HF. Data are presented as mean ± SEM. *P < 0.05, **P < 0.01, ***P < 0.001, ****P < 0.0001

Then, we also found increased m6A methylation in TAC mice by dot blot, compared with sham mice (Supplementary Fig. S1). We further investigated the driving factors behind the significantly altered m6A patterns in HF. The expression of m6A enzymes rarely changed at 1, 3, and 7 days post-TAC. Most of their expression levels decreased at 2 weeks post-TAC and further decreased at 4 and 8 weeks post-TAC (Supplementary Fig. S2a–e and Fig. 1f). The most significant decrease was observed in the mRNA expression of *Fto*. Because the levels of m6A writers were also decreased after TAC, these findings suggested FTO downregulation as the major contributor to the increased m6A levels post TAC. Consistently, the FTO protein expression levels were also significantly decreased at 8 weeks post-TAC (Fig. 1g and Supplementary Fig. S2f). To evaluate FTO expression in HF patients, the expression profiles of 13 failing and 15 non-failing hearts (GDS2206)^3^ from the Gene Expression Omnibus database were analyzed. HF patients also exhibited decreased FTO expression (Supplementary Fig. S2g).

To investigate the potential roles of FTO in HF, gain-of-function experiments by overexpressing FTO by AAV9 were performed (Supplementary Fig. S3a, b). Compared with the TAC group, FTO overexpression decreased the high m6A methylation induced by TAC (Supplementary Fig. S3c, d). FTO overexpression had no effect on cardiac function at 2 weeks post-TAC (Supplementary Fig. S3e, f). However, FTO overexpression significantly attenuated cardiac dysfunction at 4 and 8 weeks post-TAC (Fig. 1h, i and Supplementary Fig. S3g, h). FTO overexpression also attenuated left ventricular hypertrophy and enlargement post-TAC (Supplementary Table S2). Moreover, TAC + aavFTO mice exhibited significantly improved exercise endurance compared with TAC mice at 8 weeks post-TAC (Supplementary Fig. S3i). FTO overexpression had no impact on body weight in TAC mice (Supplementary Fig. S3j); however, it did significantly decrease the heart weight-to-tibia length ratio (Supplementary Fig. S3k). Furthermore, FTO overexpression did not affect the wet-to-dry lung ratio in TAC mice (Supplementary Fig. S3l). Cardiac fibrosis and cardiac hypertrophy were ameliorated in TAC + aavFTO mice (Fig. 1j and Supplementary Fig. S4a, b). In vivo cardiac glucose uptake was measured by micropositron emission tomography–computed tomography (micro-PET/CT) at 8 weeks post-TAC, and ^18^F-FDG uptake was significantly increased in TAC + aavFTO mice as compared to TAC mice (Fig. 1k and Supplementary Fig. S5). Transmission electron microscopy showed that FTO overexpression ameliorated the mitochondrial structure disorder observed in TAC mice (Supplementary Fig. S6a, b). These data suggest that FTO overexpression ameliorates cardiac dysfunction in TAC mice. However, knockdown of FTO showed opposite phenotypes (Supplementary Fig. S7a–k and S8a–c, Fig. 1l, m, and Supplementary Table S2).

Since MeRIP-seq examination revealed that the m6A modifications mainly occurred in mRNAs of metabolic pathways, we thus explored the effect of FTO knockdown on cardiac metabolism parameters. Significantly increased glucose uptake was observed by micro-PET/CT in the TAC mice, which was decreased by FTO knockdown (Fig. 1n and Supplementary Fig. S9). Next, mitochondrial structure was observed by transmission electron microscopy. Low mitochondrial–matrix electron densities, disarrayed cristae, and clustered mitochondria were demonstrated in cardiomyocytes from TAC mice and these changes were further exacerbated by FTO knockdown (Supplementary Fig. S10a, b). Taken together, our data indicated that FTO knockdown exacerbated cardiac dysfunction and remodeling, cardiac mitochondrial structure changes, and decreased cardiac energy supply post-TAC.

To further evaluate the metabolic effects of FTO in vitro, we generated three adenoviral lines for FTO knockdown in isolated primary cardiomyocytes. The strongest knockdown efficiency was confirmed in the #2 adenoviral line, which was therefore used for subsequent experiments (Supplementary Fig. S11a). Decreased FTO expression was evidenced after angiotensin II (Ang II) stimulation (Supplementary Fig. S11b, c), which mimicked the expression pattern of FTO in TAC mouse model. Extracellular acidification rate indicated that Ang II stimulation increased glycolysis in cardiomyocytes. However, FTO knockdown reduced the glycolytic capacity of cardiomyocytes (Adv-siFTO #2 in Fig. 1o, p, Adv-siFTO #1 in Supplementary Fig. S12a, b). Additionally, decreased ATP levels were detected after Ang II stimulation, which were further decreased after FTO knockdown (Fig. 1q). These metabolic alterations after Ang II stimulation were similar to those observed in the TAC-induced HF mouse model, in which ATP production was decreased despite increased glycolysis during HF development.^4^ These findings suggest an important role of FTO in cardiomyocyte metabolic homeostasis.

Since disturbances in ATP generation may directly affect contractile function,^5^ cardiomyocyte contractile function was evaluated after FTO knockdown. Single-cell-level mechanics were measured in cardiomyocytes with FTO knockdown after Ang II stimulation (Supplementary Fig. S13a–d). As expected, the cardiomyocyte contractile and relengthening profiles were reduced after Ang II stimulation and were further reduced after FTO knockdown (Supplementary Fig. S13b, c). Therefore, FTO knockdown disrupted the glycolytic capacity and ATP production after Ang II stimulation in cardiomyocytes, responsible for the aggravated contractile dysfunction. Then, in vitro gain-of-function assays were conducted to validate the benefits of FTO overexpression at the cellular level. FTO overexpression improved the reduced ATP production (Supplementary Fig. S14c) by further upregulating the increased glycolytic capacity (Supplementary Fig. S14a, b) in primary cardiomyocytes stimulated by Ang II.

KEGG analysis revealed that m6A of five glycolysis-related genes were altered post TAC (Fig. 1r). Consistently, their protein expression levels were detected in TAC mice with FTO knockdown. Furthermore, PGAM2 was significantly decreased in the TAC + KD mice compared with TAC mice (Fig. 1s and Supplementary Fig. S15b). Therefore, RNA pull-down, MeRIP-qPCR, and mRNA lifetime assays were conducted to determine the intrinsic mechanism of FTO affecting *Pgam2* mRNA. As expected, FTO was specially enriched by biotin-labeled sense *Pgam2* (Supplementary Fig. S15a), indicating that *Pgam2* interacted with FTO. Then, FTO knockdown significantly upregulated the m6A methylation of *Pgam2* mRNA (Fig. 1t) and decreased its stability (Fig. 1u), suggesting that PGAM2 might be at least partially involved in the glycolytic changes induced by FTO knockdown in the TAC mice and cardiomyocytes after Ang II stimulation.

Since alterations in cardiomyocyte glucose uptake were observed by micro-PET/CT, glucose transporter (GLUT) proteins were evaluated after FTO knockdown. FTO knockdown reduced *Glut4* expression at the transcriptional level in TAC mice (Supplementary Fig. S15c). However, MeRIP-seq did not reveal any changes in the m6A modifications of glucose uptake-related genes (Supplementary Table S1). Therefore, we speculated that FTO may regulate GLUT4 in an m6A-independent manner and thus evaluated the expression of metabolic regulatory protein kinase B (AKT). As expected, GLUT4 and phosphorylated AKT (p-AKT) protein levels were significantly downregulated in the TAC + KD mice compared with TAC mice (Fig. 1v and Supplementary Fig. S15d, e). Moreover, the expression of PGAM2, GLUT4, and p-AKT was upregulated after FTO overexpression (Supplementary Fig. S16a–d). Collectively, our finding suggests that FTO regulates glycolysis in an m6A-dependent manner and also regulates glucose uptake possibly by modulating the AKT–GLUT4 axis.

In summary, this is the first study to systematically explore the regulatory roles of m6A and FTO in cardiac metabolism using loss- and gain-of-function approaches in the TAC-induced HF mouse model. Altogether, we have identified the regulatory role of FTO in cardiac function and structure, particularly glucose metabolism (Fig. 1w). This work provides a new concept of m6A modification in cardiac metabolic homeostasis during HF and also strongly suggests FTO as a potential target for HF prevention and treatment. Considering the importance of glucose oxidation in energy supply in HF, future studies are warranted to systematically assess the role of FTO in cardiac glucose oxidation.

## Supplementary information

Supplementary material

## Data Availability

The datasets presented in this study can be found in online repositories. The names of the repository and accession number can be found at: https://www.ncbi.nlm.nih.gov/bioproject/, PRJNA707252.
