# Supplementary material for: m6A demethylase FTO attenuates cardiac dysfunction by regulating glucose uptake and glycolysis in mice with pressure overload-induced heart failure
Source: Signal Transduct Target Ther. 2021 Nov 2;6:377. doi: 10.1038/s41392-021-00699-w (PMC8563751; doi:10.1038/s41392-021-00699-w)
Supplement: Supplementary file 1 — Supplementary material [file 41392_2021_699_MOESM1_ESM.docx]

**SUPPLEMENTAL MATERIAL**

**Title:**

**m6A Demethylase** **FTO Attenuates Cardiac Dysfunction by Regulating Glucose Uptake and Glycolysis in Mice with Pressure Overload-Induced Heart Failure**

**Authors:**

Beijian Zhang^1,2,4,5#^, Hao Jiang ^1,2,4,5#^, Jian Wu^1,2,3#^, Yun Cai^1,2,4,5^, Zhen Dong ^1,2,4,5^, Yongchao Zhao^1,2,4,5^, Qinfeng Hu^1,2,3^, Kai Hu^1,2^, Aijun Sun^1,2,3,4,5*^, Junbo Ge^1,2,3,4,5*^

^#^BJ.Z., H.J. and J.W. contributed equally to this work.

^*^To whom correspondence may be addressed. Email: [jbge@zs-hospital.sh.cn](mailto:jbge@zs-hospital.sh.cn), or [sun.aijun@zs-hospital.sh.cn](mailto:sun.aijun@zs-hospital.sh.cn)

**This file includes:**

Materials and Methods

References

Figures. S1 to S16

Table S1 to S2

**SUPPLEMENTAL MATERIALS AND METHODS**

**Animal model**

Male mice (5–16 weeks old) with a C57BL/6 background were used for this study. Transverse aortic constriction (TAC) was performed by ligating the aortic arch between the brachiocephalic trunk and left common carotid artery after placement of a 27-gauge needle, as previously described^1^. Control mice (sham) underwent the same surgery without ligating the aortic arch. The numbers of mice for each experiment are indicated in the figure legends. FTO knockdown and overexpression were achieved via AAV9 (5×10^11^) tail vein injection in 5-week-old mice. At 8 weeks of age, the mice underwent TAC surgery. Mice with FTO knockdown (KD) were divided into sham, sham+KD, TAC, and TAC+KD groups for loss-of-function assays. Mice with FTO overexpression (aavFTO) were divided into sham, sham+aavFTO, TAC, and TAC+aavFTO groups for gain-of-function assays. All experimental procedures were approved by the animal ethics committee at Zhongshan Hospital, Fudan University, China.

**Transthoracic echocardiography**

Mice were evaluated by echocardiography under light isoflurane anesthesia (1–2%) at 2, 4, and 8 weeks post-TAC (VisualSonics Vevo 2100, Canada). Ejection fraction, fractional shortening, and ventricular chamber dimensions were measured as previously described^2^.

**Quantitative polymerase chain reaction (qPCR)**

Total RNA was extracted from heart tissues with Trizol reagent (Invitrogen, 15596-026, USA), and RNA quality and quantity were assessed using a NanoDrop 2000 spectrophotometer (Thermo-Fisher Scientific, USA). Reverse transcription was performed using the PrimeScript RT Reagent Kit (Takara, RR036A, Japan). Next, qPCR was performed with the SYBR Premix Ex Taq II (Takara, RR820A, Japan) and a CFX96 Real-Time System (Bio-Rad, USA). Relative gene expression was normalized to β-actin. The primers are listed in followings:

| Target name | Forward sequence | Reverse sequence |
| --- | --- | --- |
| Mettl3 | CTGGGCACTTGGATTTAAGGAA | TGAGAGGTGGTGTAGCAACTT |
| Mettl4 | TGGGTGGTTACTGGATCATCT | AGCAAAGCACATAGCAGGAGC |
| Mettl14 | CTGAGAGTGCGGATAGCATTG | GAGCAGATGTATCATAGGAAGCC |
| Wtap | TAGACCCAGCGATCAACTTGT | CCTGTTTGGCTATCAGGCGTA |
| Kiaa1429 | ATAGATGTGGTTCGTTTTCCGTG | CACTATGGGCTCGTACTCCC |
| Fto | TTCATGCTGGATGACCTCAATG | GCCAACTGACAGCGTTCTAAG |
| Alkbh5 | GCATACGGCCTCAGGACATTA | TTCCAATCGCGGTGCATCTAA |
| Ythdf1 | ACAGTTACCCCTCGATGAGTG | GGTAGTGAGATACGGGATGGGA |
| Ythdf2 | GAGCAGAGACCAAAAGGTCAAG | CTGTGGGCTCAAGTAAGGTTC |
| Ythdf3 | GATCAGCCTATGCCATATCTGAC | CCCCTGGTTGACTAAAAACACC |
| Ythdc1 | GTCCACATTGCCTGTAAATGAGA | GGAAGCACCCAGTGTATAGGA |
| Ythdc2 | GAAGATCGCCGTCAACATCG | GCTCTTTCCGTACTGGTCAAA |
| Glut1 | CAGTTCGGCTATAACACTGGTG | GCCCCCGACAGAGAAGATG |
| Glut2 | TCAGAAGACAAGATCACCGGA | GCTGGTGTGACTGTAAGTGGG |
| Glut3 | ATGGGGACAACGAAGGTGAC | GTCTCAGGTGCATTGATGACTC |
| Glut4 | GTGACTGGAACACTGGTCCTA | CCAGCCACGTTGCATTGTAG |
| Glut5 | CCAATATGGGTACAACGTAGCTG | GCGTCAAGGTGAAGGACTCAATA |
| Glut6 | AACCGAGGGACTCGACTATGA | CAAGGCATACCCAAAGCTGAA |
| Glut8 | CCCTTCGTGACTGGCTTTG | TGGGTAGGCGATTTCCGAGAT |
| Glut9 | TTGCTTTAGCTTCCCTGATGTG | GAGAGGTTGTACCCGTAGAGG |
| Glut10 | GCCTGACCTTCGGATATGAGC | TGCCATAGCAGTCAATGAGGA |
| Glut12 | AGGTCCCAGCATGTTTACGTT | GGGCTAATAGCGTTCTGATCTG |
| Pgam2 | TGGAACCAAGAGAACCGTTTC | TGGCATCTTTGATAGCGGTGG |

**Immunoblot assay**

Proteins were isolated from heart tissues and primary cardiomyocytes from WT mice with RIPA buffer on ice. Total protein lysates were separated by sodium dodecyl sulfate-polyacrylamide gel electrophoresis (SDS-PAGE) and transferred to polyvinylidene fluoride membranes. The membranes were blocked with 5% bovine serum albumin and individually probed with primary antibodies overnight at 4℃. After washing three times with tris-buffered saline with 0.1% Tween 20 (TBST), the membranes were incubated with horseradish peroxidase (HRP)-coupled anti-rabbit or anti-mouse secondary antibodies for 1 hour at room temperature. Proteins were detected using chemiluminescence reaction substrate (Luminata Forte, Millipore, USA) and the ChemiDoc Imaging System (Bio-Rad, CA, US). The immunoblots were quantified by ImageJ software (1.50i，USA) after normalization to β-actin levels. The antibodies used in this study are shown in followings:

| WB Antibody | Item No. | WB Antibody | Item No. |
| --- | --- | --- | --- |
| FTO | ab92821 (Abcam) | β-actin | KC5A03 (KANG CHEN, China) |
| PGAM2 | ab187147 (Abcam) | TPI1 | ab96696 (Abcam) |
| PGM2 | ab151746 (Abcam) | DLD | ab133551 (Abcam) |
| ALDOB | ab75751 (Abcam) | GLUT4 | 2213 (CST) |
| p-AKT (Ser 473) | 4060s (CST) | AKT | 4619s (CST) |

**Methylated RNA immunoprecipitation sequencing (MeRIP-seq)**

Total RNA was extracted from heart tissues (TAC and sham) with Trizol reagent, and polyA^+^ RNA was enriched from total RNA with oligo(dT) magnetic beads. The polyA^+^ RNA was fragmented into lengths of ~100 nucleotides with RNA fragmentation buffer (Millipore Sigma, USA). Part of the fragmented RNA was then used for m6A enrichment/immunoprecipitation and as the input to construct the conventional transcriptome sequencing library. After the fragmented RNA was enriched for m6A, the sequencing libraries were constructed and sequenced using the Illumina Hiseq X Ten sequencing platform.

**Dot-blots assay**

Total RNA was extracted with Trizol reagent (Invitrogen) and the quality and quantity of RNA were assessed by NanoDrop 2000 (Thermo Fisher Scientific). The RNA was diluted with DEPC to the final concentration of 100 ng/μL. 2 μL of RNA was denatured by heating at 95 °C for 5 min, then chilling on ice immediately to prevent the secondary structure of mRNA from re-forming. Next, RNA was spotted on Biodyne Nylon Transfer Membranes (Pall) and cross-linked to the membrane by UV using HL-2000 HybriLinker (UVP). The membranes were blocked with 5% BSA (bull serum albumin) in TBST (tris buffered saline tween) and the global m6A level was detected by using the m6A-specific antibody (CST, 1:1000), then the membranes were methylene blue stained as loading control.

**Adult mouse cardiomyocyte isolation, adenoviral infection, angiotensin II stimulation, and mechanics analyses**

For *in vitro* assays to assess cardiomyocyte metabolism and mechanics, adult mouse cardiomyocytes were isolated, as previously described^3^. Briefly, hearts were perfused with ethylene-diamine-tetraacetic acid (EDTA) and perfusion buffers and were then digested with a digestion buffer containing protease XIV, collagenase II, and collagenase IV. Digested hearts were then torn into 1-mm^3^ pieces. Isolated cardiomyocytes were collected by gravity sedimentation after filtration through a 100-µm filter*.* Adenoviral infection was used to achieve *in* vitro FTO overexpression and knockdown. The FTO shRNA adenovirus (pDKD-CMV-eGFP-U6-FTO shRNA), FTO overexpression adenovirus (pAdeno-EF1A(S)-mNeonGreen-CMV-FTO-3FLAG), and the respective negative controls were obtained from Obio Technology Corp. (Shanghai, China). After adenoviral infection for 24 hours, the cardiomyocytes were treated with 1,000 nM of angiotensin II (Ang II; Sigma-Aldrich, A9525, USA) for 48 hours. Mechanics analyses were performed using the IonOptix SoftEdge System, as previously described^2^. Briefly, cardiomyocytes were treated with a calcium buffer containing 130 mM NaCl, 5.4 mM KCl, 10 mM HEPES, 1.8 mM CaCl_2_, 0.5 mM MgCl_2_, and 10 mM glucose (pH 7.4). Viable, rod-shaped cardiomyocytes were placed in the center of the microscope field. Contractile and relengthening profiles of cardiomyocytes were assessed according to the following parameters: peak shortening (PS; representing cardiomyocyte length after electrical stimulation and normalization to the resting cell length and indicating the peak ventricular contractility), maximal velocity of shortening (-dL/dt) and relengthening (+dL/dt), and time-to-90% relengthening (TR90; representing the duration of relaxation).

**ATP assay**

ATP was detected using the Enhanced ATP Assay Kit (Beyotime Biotechnology, S0027, China), according to the manufacturer’s instructions. Briefly, the collected cells were lysed with lysis buffer and then centrifuged at 12,000 ×g for 5 minutes at 4℃. ATP was detected in the supernatants after adding a working buffer using a chemiluminescence apparatus (Thermo-Fisher Scientific, USA). ATP concentrations were normalized to the corresponding protein concentrations.

**Bioenergetics assay**

Extracellular acidification rates (ECARs) were determined by the XFe96 Extracellular Flux Analyzer (Seahorse Bioscience, USA), as previously described^2^. Briefly, isolated adult mouse cardiomyocytes were seeded into the XFe 96-well detection plate at a density of 2×10^3^ cells/well, and the four corners of the detection plate were used as blank controls. After the cells were treated with adenovirus and angiotensin II, as described above, the ECARs were detected.

**Micro-PET/CT imaging**

Mice were fasted for 8 hours before the examination and were anesthetized by isoflurane. Then, 200 μCi of ^18^F-labeled fluorodeoxyglucose (FDG) (obtained from the Department of Nuclear Medicine, Fudan University Shanghai Cancer Center) was intraperitoneally injected. After 1 hour, PET/CT scanning and imaging were performed using the Inveon micro-PET/CT System, and analyses were conducted using the Inveon Research Workplace software (Siemens Medical Solution, California, USA).

**Histological analyses**

Paraffin-embedded or frozen heart tissues were sectioned at a thickness of 5 μm. The sections were then deparaffinized and stained with different dyes. Hematoxylin and eosin staining was used to visualize the structure of myocardial cells and the intercellular stroma. Masson’s trichrome staining was performed to detect fibrosis. Wheat germ agglutinin staining was used to analyze cardiac hypertrophy.

For immunofluorescence staining, paraffin sections were incubated with GLUT4 (Abcam, ab654, USA) primary antibody overnight, followed by incubation with a suitable fluorophore-conjugated secondary antibody for 1 hour.

**Transmission electron microscopy**

Heart tissues were cut into 1-mm^3^ pieces and then fixed in 2.5% glutaraldehyde. Tissues were dehydrated in increasing gradients of ethanol solution (50–90%) and were then transferred to acetone solution and embedding solution. After fixation and embedding, the tissues were sectioned at a thickness of 50 nm. The sections were stained with 3% uranium acetate and lead citrate. Mitochondrial morphology and structure were observed under a CM-120 transmission electron microscope (Philips, The Netherlands). At least 10 fields were analyzed for each mouse.

**Methylated RNA immunoprecipitation-qPCR (MeRIP-qPCR)**

MeRIP-qPCR was performed to validate m6A methylation alterations in a certain target gene, as previously described^4^. The Magna MeRIP m6A Kit (Millipore, 17-10499, USA) was used according the manufacturer’s instructions. Briefly, total RNA was extracted from the mouse heart tissues of the TAC and TAC+KD groups with Trizol reagent (Invitrogen, USA). Then, 200 μg of total RNA from each group was fragmented (~200 nucleotides), incubated with m6A antibody (Abcam, 56593S, USA), and then pulled down using Protein A Dynabeads (Invitrogen, USA). After digesting the enriched RNA/antibody complex with protease, the m6A antibody was digested and the remaining RNA was used for routine reverse transcription and qPCR.

**RNA pull-down**

RNA pull-down assay was performed by using the Magnetic RNA‑Protein Pull‑Down kit (Thermo Fisher), as described previously^5^. Brieﬂy, cells were lysed then centrifuged at 14,000 g for 10 min at 4˚C to pellet the cell debris and obtain cell lysates (cell supernatants). Cell lysates (25 µL per IP reaction) were co‑incubated with nucleic acid-compatible streptavidin magnetic beads (Thermo Fisher) combined with biotinylated sense or antisense *Pgam2*. Proteins of the RNA-protein complexes were eluted from the magnetic beads by boiling, and FTO protein expression was examined as aforementioned via western blotting.

**Life-time assay for mRNA**

FTO knockdown was achieved by adenoviral infection (Ad-shFTO) in isolated primary adult mouse cardiomyocytes. After 24 hours of infection, the cells were treated with actinomycin D (Sigma, A9415) to inhibit transcription. Then, total RNA was extracted at 0, 2, and 4 hours after transcriptional inhibition and was subsequently subjected to reverse transcription and qPCR. The relative half-life of *Pgam2* mRNA was determined, as previously described^6^. The mRNA degradation rate (K) was estimated using the following equation: ln(C_t_/C_0_)=-Kt, where t is the transcriptional inhibition time (hours), and C_t_ and C_0_ represent the mRNA quantity at time t and time 0, respectively. When mRNA decay reached 50% (i.e., C_t_/C_0_=1/2), the equation below was used to calculate the mRNA half-life (t_1/2_): ln(1/2)=-Kdecay t(1/2), where t(1/2)=ln2/K.

**Statistics**

Statistical analyses were performed using GraphPad Prism 7.0. Data were expressed as the mean ± SEM. A Shapiro-Wilk test was performed to determine normality. Normal distributions were compared using an unpaired Student’s t-test (between two groups), one-way ANOVA followed by Tukey’s post hoc test, or two-way ANOVA followed by Bonferroni’s post hoc test (among three or more groups). Comparisons between non-normal distributions were made using a Mann-Whitney U test or Kruskal-Wallis H test. Statistical significance was indicated by *P*<0.05.

**Discussion of the differences between our study and related literatures**

Mathiyalagan *et al*. explored the effect of FTO on heart failure after myocardial infarction by using heart samples from patients with myocardial infarction, myocardial infarction tissue from pig and mouse ^7^. Similar to our study, they also found that FTO is down regulated in infarcted tissues. Overexpression of FTO reduced infarct size and myocardial fibrosis, and enhanced cardiac contractile function, thus promoting myocardial repair after myocardial infarction^7^. The difference is that we used the heart failure model induced by TAC, and we focused on the influence of FTO on energy metabolism (specifically glucose metabolism) in TAC-induced heart failure. Berulava *et al*. constructed FTO-cKO mice and found that FTO knockout aggravated TAC-induced cardiac dysfunction^8^. However, they only evaluated the cardiac function in mice by echocardiography, and other phenotypes like cardiac remodeling and underlying mechanisms were not detected. In our study, we systematically evaluated the effects of FTO on cardiac remodeling and energy metabolism in heart failure through FTO knockdown and overexpression *in vivo* and *in vitro*. At the same time, we explored the regulatory mechanism of FTO on myocardial energy metabolism through RNA pull-down, MeRIP-qPCR and detecting half-life of target mRNA. Consistent with our results, Dorn *et al.* also found that m6A methylation was significantly increased in cardiomyocytes after hypertrophic stimulation, and m6A methylase METTL3 governs cardiac homeostasis and hypertrophy^9^. Moreover, METTL3 was also found to be involved in hypoxic pulmonary hypertension by regulating the proliferation and migration of pulmonary artery smooth muscle cells^10^. It is to note that there were different responses post FTO-KD *in vivo* and *in vitro*. Strong effects of FTO-KD were observed *in vitro*, while in sham mice, knockdown of FTO did not induce HF phenotype, future studies are warranted to address this issue.

**SUPPLEMENTAL REFERENCES**

1. Li X, Weng X, Shi H, Gao R, Wang P, Jia D, Zhang S, Dong Z, Sun X, Yang J, Wang Z, Liu R, Li Y, Qiu Z, Hu K, Sun A and Ge J. Acetaldehyde dehydrogenase 2 deficiency exacerbates cardiac fibrosis by promoting mobilization and homing of bone marrow fibroblast progenitor cells. *Journal of molecular and cellular cardiology*. 2019;137:107-118.

2. Jiang H, Jia D, Zhang B, Yang W, Dong Z, Sun X, Cui X, Ma L, Wu J, Hu K, Sun A and Ge J. Exercise improves cardiac function and glucose metabolism in mice with experimental myocardial infarction through inhibiting HDAC4 and upregulating GLUT1 expression. *Basic research in cardiology*. 2020;115:28.

3. Ackers-Johnson M, Li PY, Holmes AP, O'Brien SM, Pavlovic D and Foo RS. A Simplified, Langendorff-Free Method for Concomitant Isolation of Viable Cardiac Myocytes and Nonmyocytes From the Adult Mouse Heart. *Circulation research*. 2016;119:909-20.

4. Liu J, Eckert MA, Harada BT, Liu SM, Lu Z, Yu K, Tienda SM, Chryplewicz A, Zhu AC, Yang Y, Huang JT, Chen SM, Xu ZG, Leng XH, Yu XC, Cao J, Zhang Z, Liu J, Lengyel E and He C. m(6)A mRNA methylation regulates AKT activity to promote the proliferation and tumorigenicity of endometrial cancer. *Nature cell biology*. 2018;20:1074-1083.

5. Qin B, Dong M, Wang Z, Wan J, Xie Y, Jiao Y and Yan D. Long non‑coding RNA CASC15 facilitates esophageal squamous cell carcinoma tumorigenesis via decreasing SIM2 stability via FTO‑mediated demethylation. *Oncology reports*. 2021;45:1059-1071.

6. Chen CY, Ezzeddine N and Shyu AB. Messenger RNA half-life measurements in mammalian cells. *Methods in enzymology*. 2008;448:335-57.

7. Mathiyalagan P, Adamiak M, Mayourian J, Sassi Y, Liang Y, Agarwal N, Jha D, Zhang S, Kohlbrenner E, Chepurko E, Chen J, Trivieri MG, Singh R, Bouchareb R, Fish K, Ishikawa K, Lebeche D, Hajjar RJ and Sahoo S. FTO-Dependent N(6)-Methyladenosine Regulates Cardiac Function During Remodeling and Repair. *Circulation*. 2019;139:518-532.

8. Berulava T, Buchholz E, Elerdashvili V, Pena T, Islam MR, Lbik D, Mohamed BA, Renner A, von Lewinski D, Sacherer M, Bohnsack KE, Bohnsack MT, Jain G, Capece V, Cleve N, Burkhardt S, Hasenfuss G, Fischer A and Toischer K. Changes in m6A RNA methylation contribute to heart failure progression by modulating translation. *European journal of heart failure*. 2020;22:54-66.

9. Dorn LE, Lasman L, Chen J, Xu X, Hund TJ, Medvedovic M, Hanna JH, van Berlo JH and Accornero F. The N(6)-Methyladenosine mRNA Methylase METTL3 Controls Cardiac Homeostasis and Hypertrophy. *Circulation*. 2019;139:533-545.

10. Qin Y, Qiao Y, Li L, Luo E, Wang D, Yao Y, Tang C and Yan G. The m(6)A methyltransferase METTL3 promotes hypoxic pulmonary arterial hypertension. *Life sciences*. 2021;274:119366.

**Supplementary figures**

Fig. S1


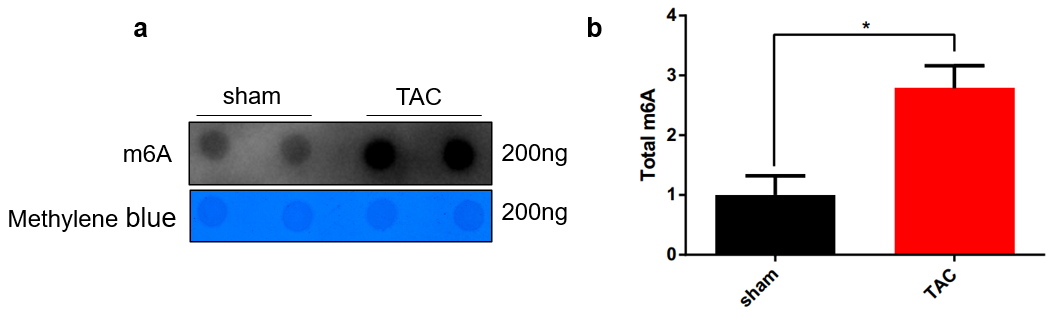


**Figure S1. Increased total level of m6A methylation in TAC mice by dot blots.**

**a.** The MeRIP-seq data were further confirmed by RNA m6A dot blots. **b.** Quantification of total m6A methylation level. n=4/group. The data are presented as the mean ± SEM. **P*<0.05, as compared with sham by Student’s t-test.

Fig. S2


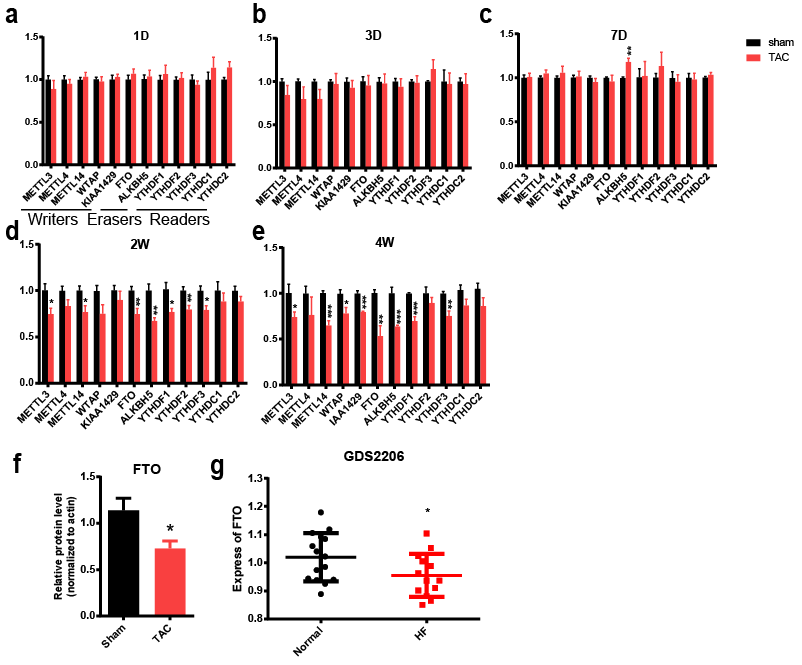


**Figure S2. Decreased FTO expression in mouse and human failing hearts.**

**a–e.** Quantification of m6A methyltransferase (*Mettl3, Mettl4, MEettl14, Wtap,* and *Kiaa1429*), demethylase (*Fto* and *Alkbh5*), and methyl-specific binding protein (*Ythdf1-3, Ythdc,1* and *Ythdc2*) mRNA levels in left ventricles of mice at 1, 3, and 7 days (D) and 2, and 4 weeks (W) post-TAC (n=5–6/group). **f.** FTO/β-actin expression at 8 weeks post-TAC (n=6/group). **g.** FTO expression in HF patients and healthy controls (n=13 HF patients and 15 controls). The data are presented as the mean ± SEM. **P*<0.05, ***P*<0.01, ****P*<0.001, *****P*<0.0001, as compared with sham or control by Student’s t-test.

Fig. S3


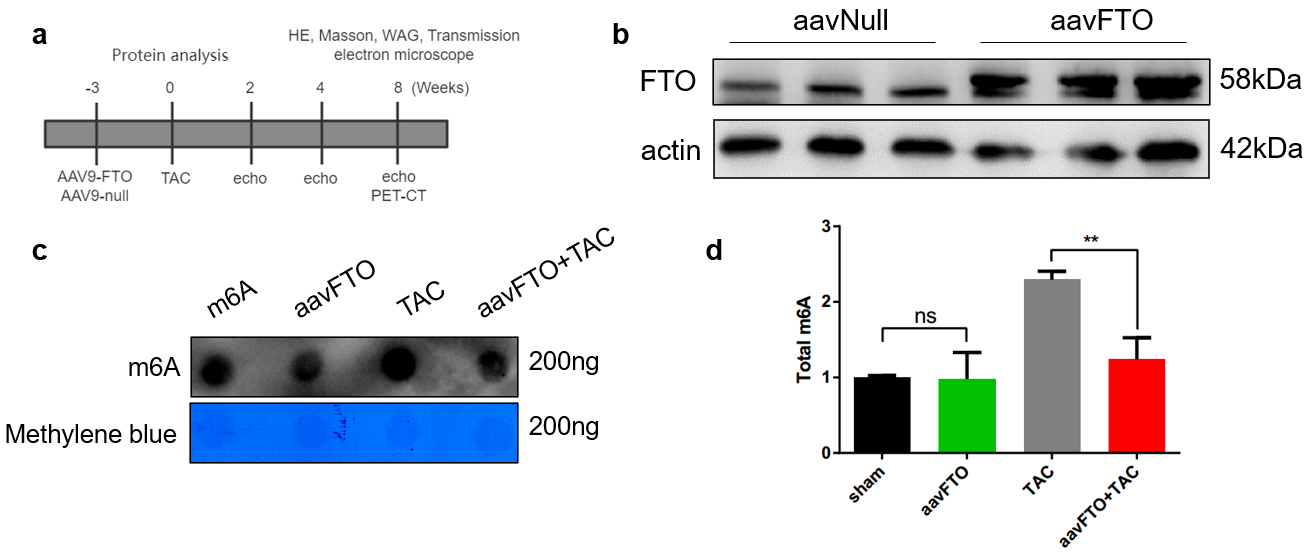


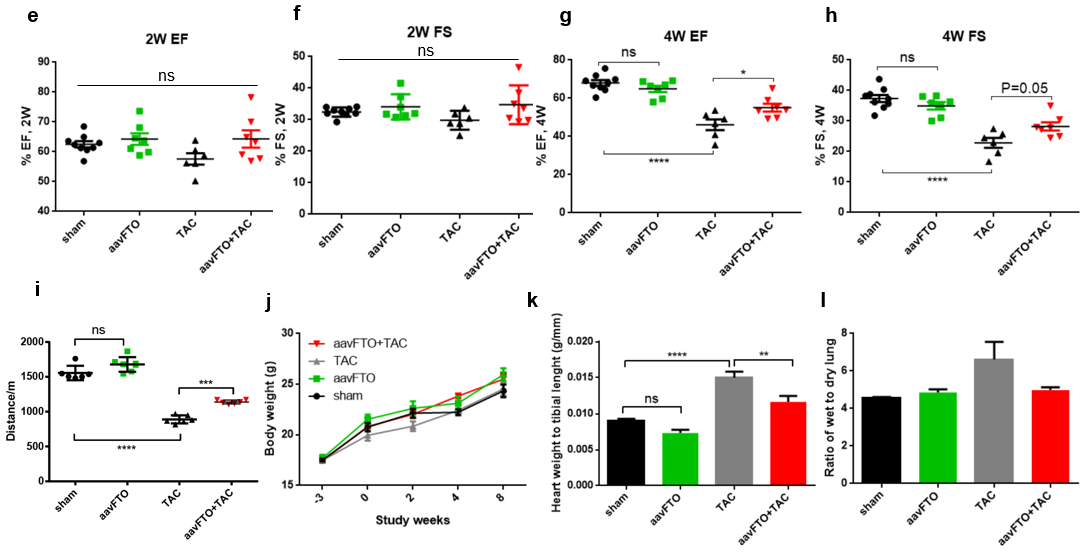


**Figure S3. Cardiac function and body weight measurements in mice with AAV9-mediated FTO overexpression post-TAC.** **a.** Study design of FTO overexpression. **b.** Immunoblots of FTO protein levels after AAV9-mediated FTO overexpression (aavFTO). **c–d.** Dot blot and quantification of total m6A methylation after FTO overexpression (n=3/group). **e–f.** Echocardiographic ejection fraction (EF) and fractional shortening (FS) measurements at 2 weeks (W) post-TAC (n=6–9/group). **g–h.** EF and FS measurements at 4 weeks post-TAC (n=6–9/group). **i.** Exercise endurance (running distance) of mice at 8 weeks post-TAC (n=6–7/group). **j.** Body weight measurements at -3, 0, 2, 4, and 8 weeks post-TAC (n=6/group). **k.** Heart weight measurements (n=6/group). **l.** Wet to dry lung ratios (n=6/group). Data are presented as the mean ± SEM. **P*<0.05, ***P*<0.01, ****P*<0.001, *****P*<0.0001 by two-way ANOVA followed by Bonferroni’s post hoc test.

Fig. S4


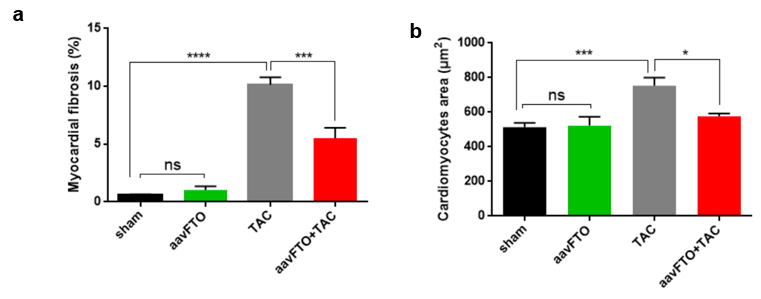


**Figure S4.** The effect of FTO overexpression on cardiac remodeling. **a.** Quantitation of myocardial fibrosis (n=4/group). **b.** Quantitation of cardiomyocyte area (n=6/group). Data are presented as the mean ± SEM. **P*<0.05, ***P*<0.01, ****P*<0.001, *****P*<0.0001 by two-way ANOVA followed by Bonferroni’s post hoc test.

Fig. S5


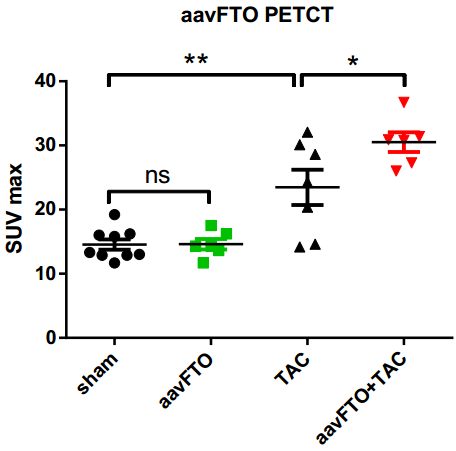


**Figure S5.** The quantification of cardiac ^18^F-FDG uptake by micro-PET/CT in FTO-overexpressed mice (n=6–9/group). Data are presented as the mean ± SEM. **P*<0.05, ***P*<0.01 by two-way ANOVA followed by Bonferroni’s post hoc test.

Fig. S6


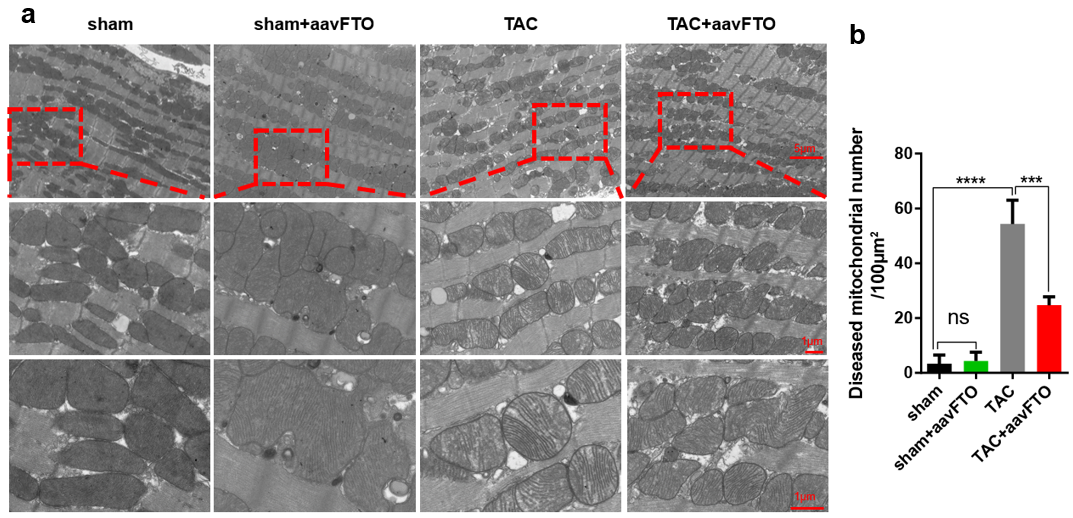


**Figure S6.** **Transmission electron microscopy of mitochondrial structure after AAV9-mediated FTO overexpression** **at 8 weeks post-TAC**. **a.** Mitochondrial structure by transmission electron microscopy, 3000×(upper panel), 8000×(middle panel), 1,5000×(bottom panel). **b.** Quantification of impaired mitochondrial number (n=3/group). Data are presented as the mean ± SEM. ***P*<0.01, *****P*<0.0001 by two-way ANOVA followed by Bonferroni’s post hoc test.

Fig. S7


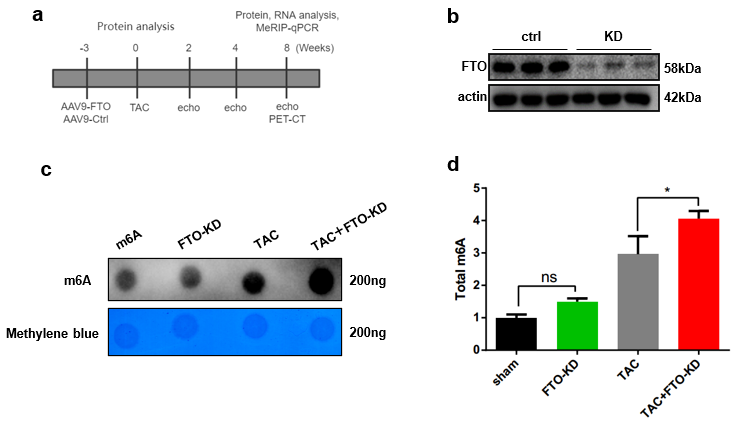


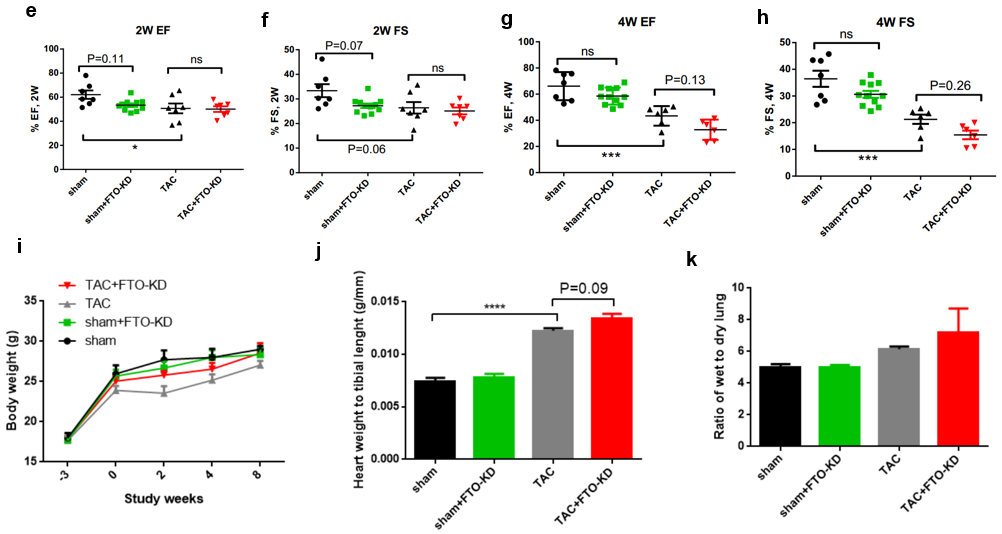


**Figure S7. Cardiac function and body weight measurements in mice with FTO knockdown post-TAC.** **a**. Design of *in vivo* FTO knockdown study. **b.** Immunoblots of FTO protein levels after AAV9-mediated FTO knockdown (KD). **c–d.** Dot blot and quantification of total m6A methylation after FTO knockdown (n=3/group). **e–f.** Echocardiographic ejection fraction (EF) and fractional shortening (FS) measurements at 2 weeks (W) post-TAC (n=6–10/group). **g–h.** EF and FS measurements at 4 weeks post-TAC (n=6–10/group). **i.** Body weight of mice at different time points (n=6/group). **j.** Heart weight measurements (n=6/group). **k.** Wet to dry lung ratios (n=6/group). Data are presented as the mean ± SEM. **P*<0.05, ***P*<0.01, ****P*<0.001, *****P*<0.0001 by two-way ANOVA followed by Bonferroni’s post hoc test.

Fig. S8


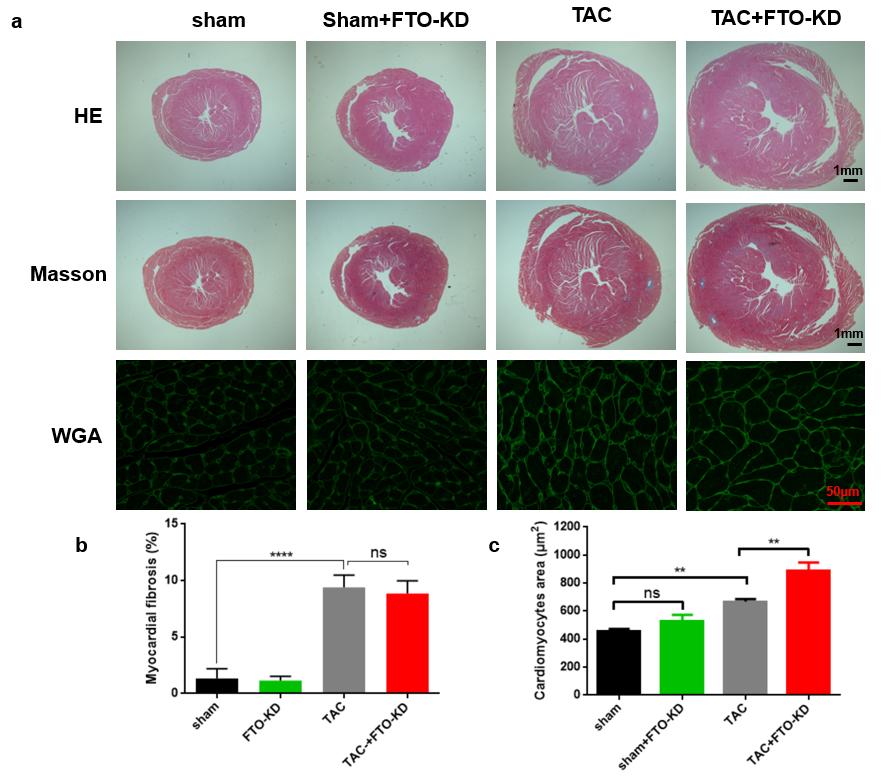


**Figure S8. Immunohistochemistry (IHC) analysis of hearts with FTO knockdown at 8 weeks post-TAC. a.** Representative images of hematoxylin and eosin (HE), Masson’s trichrome, and wheat germ agglutinin (WGA) staining. **b.** Quantitation of myocardial fibrosis (n=4/group). **c.** Quantitation of cardiomyocyte area (n=6/group). Data are presented as the mean ± SEM. **P*<0.05, ***P*<0.01, ****P*<0.001, *****P*<0.0001 by two-way ANOVA followed by Bonferroni’s post hoc test.

Fig. S9


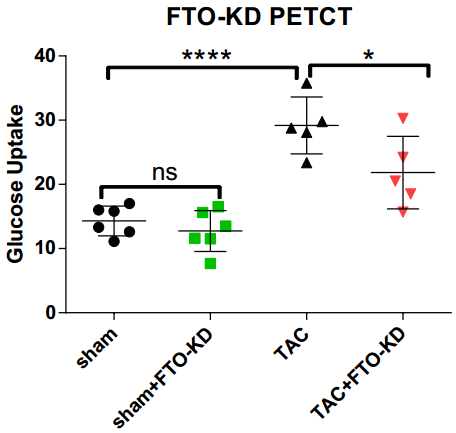


**Figure S9.** **The quantification of cardiac ^18^F-FDG uptake by micro-PET/CT in FTO-knockdown mice (n=5–6/group).** Data are presented as the mean ± SEM. **P*<0.05, *****P*<0.0001 by two-way ANOVA followed by Bonferroni’s post hoc test.

Fig. S10


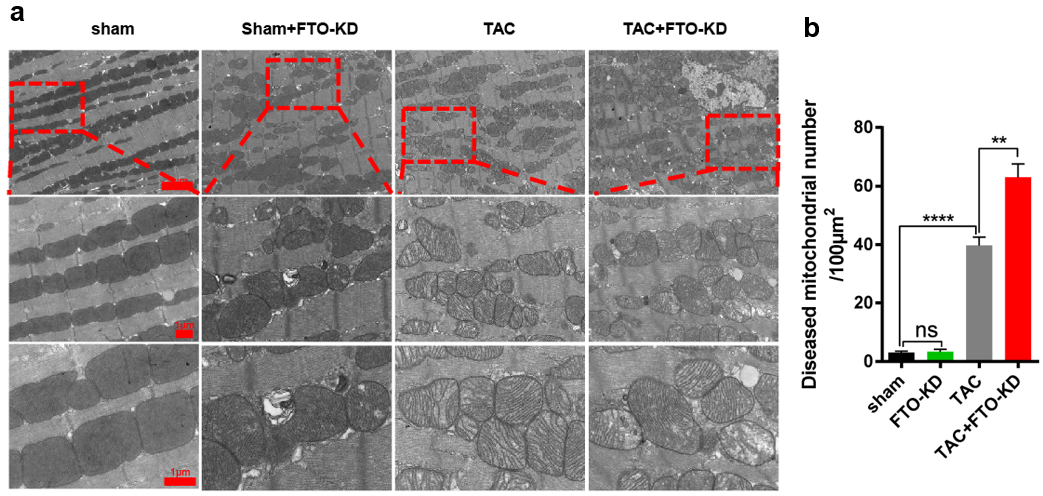


**Figure S10. Structure of impaired mitochondria after FTO knockdown at 8 weeks post-TAC. a.** Mitochondrial structure by transmission electron microscopy, 3000×(upper panel), 8000×(middle panel), 1,5000×(bottom panel). **b.** Statistics of impaired mitochondrial number (n=3/group). Data are presented as the mean ± SEM. ***P*<0.01, *****P*<0.0001 by two-way ANOVA followed by Bonferroni’s post hoc test.

Fig. S11


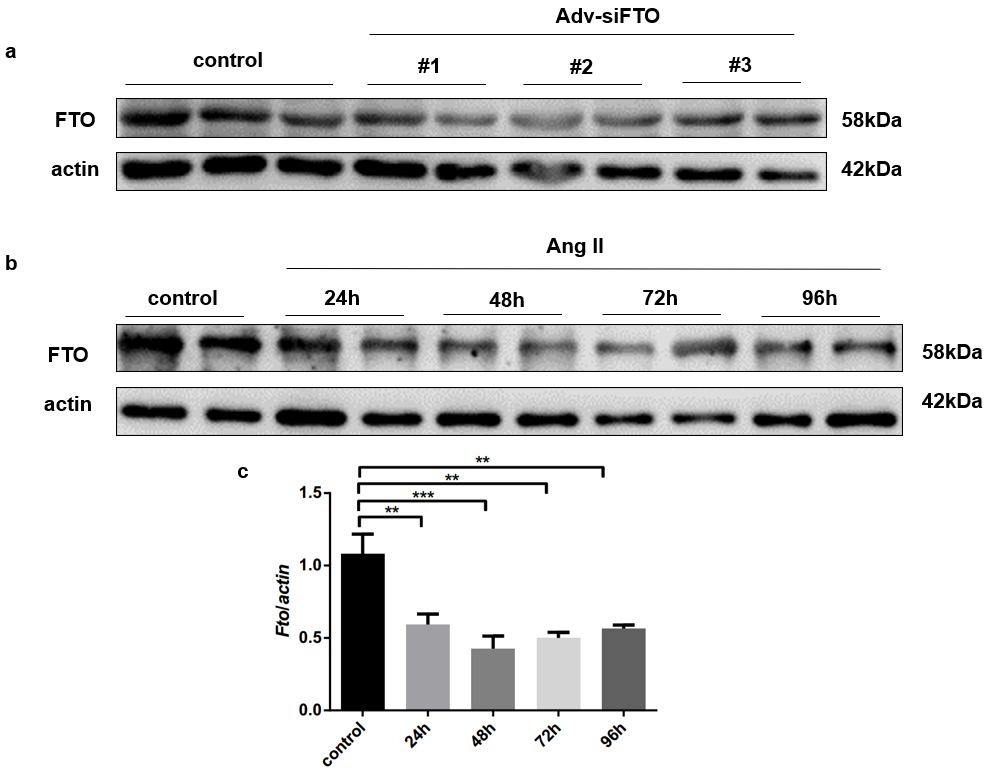


**Figure S11. Protein and RNA analysis in isolated adult mouse primary cardiomyocytes.** **a–b.** Immunoblots of FTO protein levels after adenovirus-mediated FTO knockdown (Adv-siFto) and at different time points after angiotensin II (Ang II) stimulation. c. RT-PCR of *Fto* after Ang II stimulation (n=3/group). The data are presented as the mean ± SEM. **P*<0.05, ***P*<0.01, ****P*<0.001, *****P*<0.0001, as compared with control by Student’s t-test.

Fig. S12


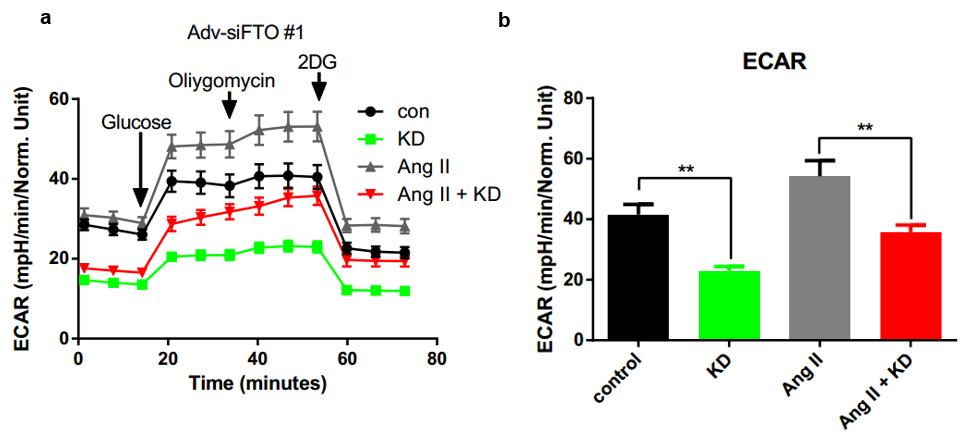


**Figure S12. Knockdown of FTO by Adv-siFTO #1 in primary cardiomyocytes reduced glycolytic capacity. a.** Extra cellular acidification rates (ECARs) of isolated cardiomyocytes using the Seahorse XF Analyzer. **b.** Glycolytic function (n=13–17/group). Data are presented as the mean ± SEM. **P*<0.05, ***P*<0.01, ****P*<0.001, *****P*<0.0001 by two-way ANOVA followed by Bonferroni’s post hoc test.

Fig. S13


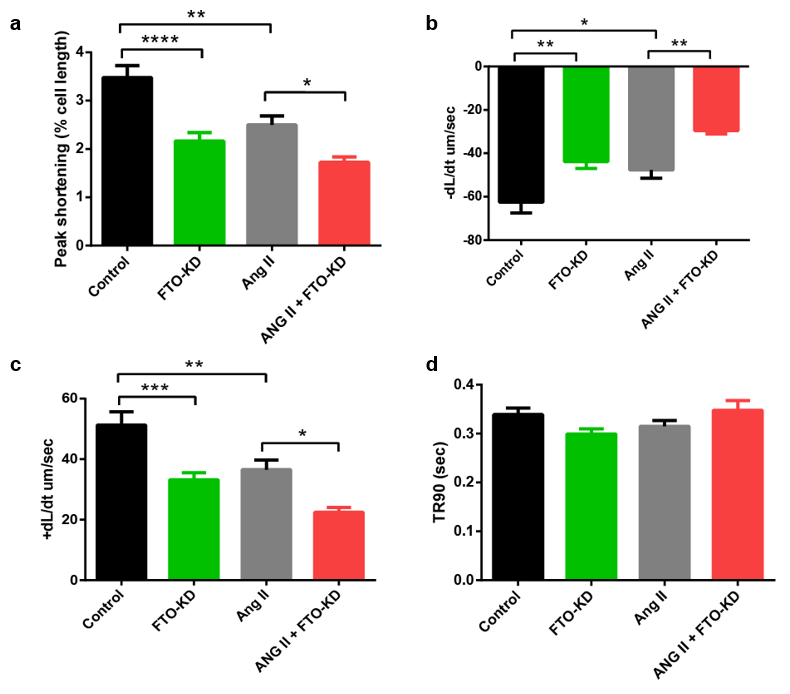


**Figure S13. FTO regulation of mechanics in primary cardiomyocytes. a–d.** Single-cell level analysis of cardiomyocyte mechanics: peak shortening (a), -dL/dt (b), +dL/dt (c), TR90 (d) (n=48–74 cells/group from 8–10 mice). Data are presented as the mean ± SEM. **P*<0.05, ***P*<0.01, ****P*<0.001, *****P*<0.0001 by two-way ANOVA followed by Bonferroni’s post hoc test.

Fig. S14


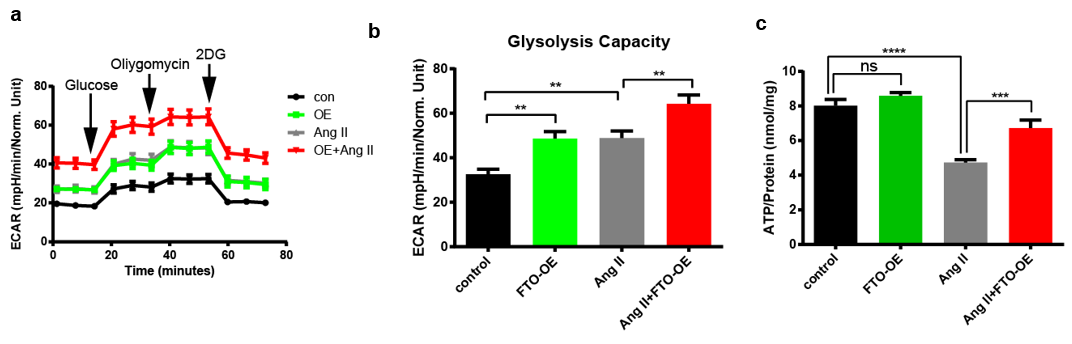


**Figure S14. Reversal of cellular changes in cardiomyocytes after angiotensin II stimulation by FTO overexpression.**

**a.** Extracellular acidification rates (ECARs) of isolated cardiomyocytes using the Seahorse XF Analyzer. **b.** Glycolytic function of isolated cardiomyocytes (n=14–17/group). **c.** ATP production (n=8/group). Data are presented as the mean ± SEM. Data are compared by two-way ANOVA followed by Bonferroni’s post hoc test. **P*<0.05, ***P*<0.01, ****P*<0.001, *****P*<0.0001.

Fig. S15


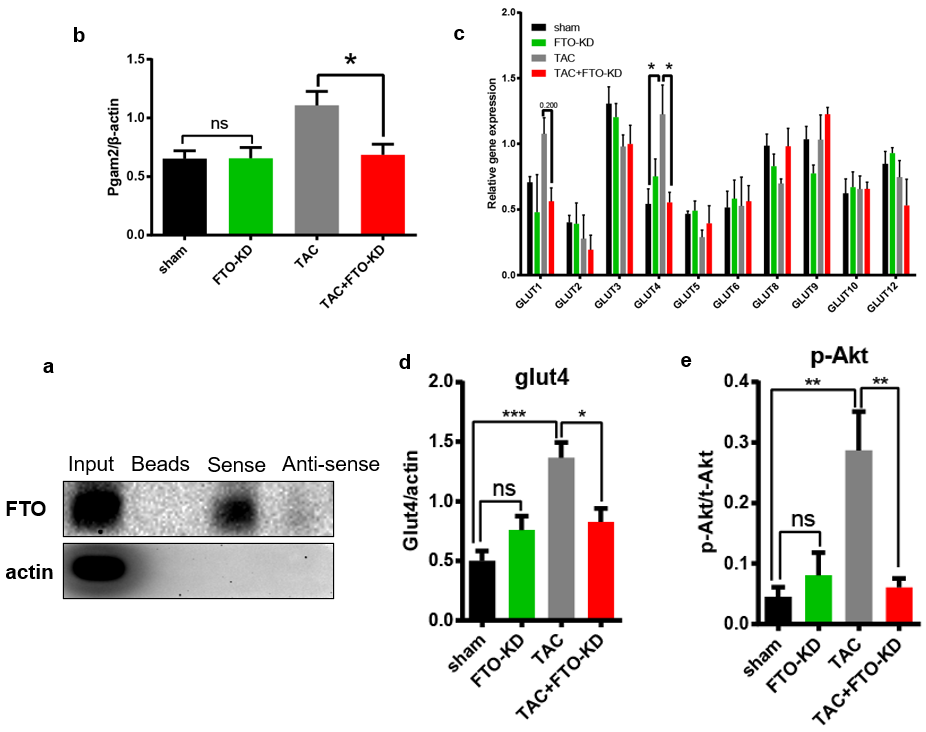


**Figure S15. FTO regulatory mechanism of cardiac glycolysis and glucose uptake.**

**a.** Immunoblotting of FTO after RNA pull-down assay with biotinylated-*Pgam2*. **b.** PGAM2 quantification (n=4/group). **c.** Analysis of GLUT family gene expression by qRT-PCR (n=3/group). **d–e.** Quantification of GLUT4 and p-AKT (n=4/group). Data are presented as the mean ± SEM. Data are compared by two-way ANOVA followed by Bonferroni’s post hoc test. **P*<0.05, ***P*<0.01, ****P*<0.001, *****P*<0.0001.

Fig. S16


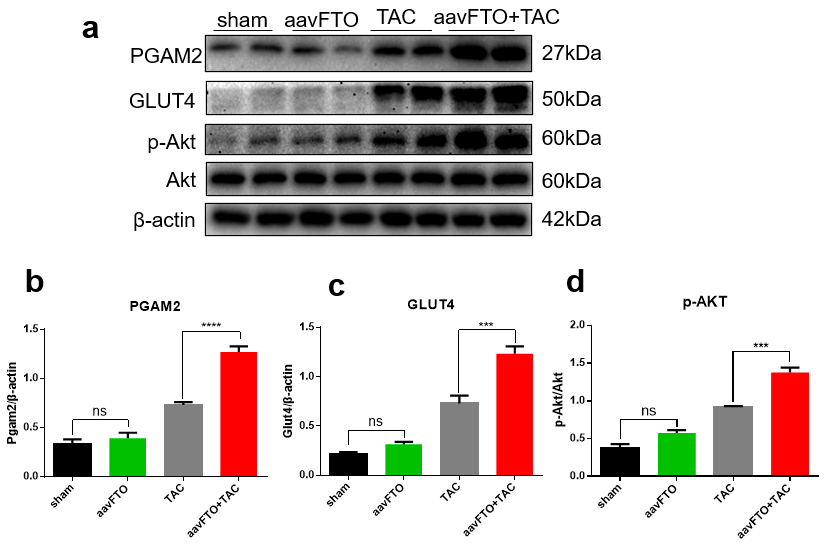


**Figure S16. FTO overexpression upregulated PGAM2 and activated AKT-GLUT4 axis.** a. Western blots of PGAM2, GLUT4 and p-AKT. b-d. Quantification of PGAM2 (b), GLUT4 (c) and p-AKT (d) expression (n=4/group). Data are presented as the mean ± SEM. Data are compared by two-way ANOVA followed by Bonferroni’s post hoc test. **P*<0.05, ***P*<0.01, ****P*<0.001, *****P*<0.0001.

**SUPPLEMENTAL TABLES**

**Supplementary Table S1. mRNAs with altered m6A methylation in HF**

| **Chr** | **Start** | **End** | **Width** | **Annotation** | **Gene Id** | **Description** |
| --- | --- | --- | --- | --- | --- | --- |
| Chr1 | 36105901 | 36106347 | 447 | 3' UTR | Hs6st1 | heparan sulfate 6-O-sulfotransferase 1 |
| Chr1 | 43533756 | 43569973 | 36218 | 3' UTR | Nck2 | non-catalytic region of tyrosine kinase adaptor protein 2 |
| Chr1 | 45339393 | 45340276 | 884 | Exon | Col3a1 | collagen, type III, alpha 1 |
| Chr1 | 59484526 | 59485220 | 695 | 3' UTR | Fzd7 | frizzled class receptor 7 |
| Chr1 | 59982507 | 59982806 | 300 | 3' UTR | Fam117b | family with sequence similarity 117, member B |
| Chr1 | 106389737 | 106392459 | 2723 | Exon | Phlpp1 | PH domain and leucine-rich repeat protein phosphatase 1 |
| Chr1 | 125872468 | 125872866 | 399 | 3' UTR | Gpr39 | G protein-coupled receptor 39 |
| Chr1 | 132507694 | 132508191 | 498 | 3' UTR | Rbbp5 | retinoblastoma-binding protein 5 |
| Chr1 | 132938557 | 132939955 | 1399 | 3' UTR | Lrrn2 | leucine-rich repeat protein 2, neuronal |
| Chr1 | 134507444 | 134507738 | 295 | 3' UTR | Rabif | RAB-interacting factor |
| Chr1 | 164280709 | 164287697 | 6989 | 3' UTR | Ccdc181 | coiled-coil domain containing 181 |
| Chr1 | 171462937 | 171463204 | 268 | 3' UTR | F11r | F11 receptor |
| Chr1 | 172317543 | 172318664 | 1122 | Exon | Igsf8 | immunoglobulin superfamily, member 8 |
| Chr1 | 172376680 | 172376929 | 250 | 5' UTR | Pigm | phosphatidylinositol glycan anchor biosynthesis, class M |
| Chr1 | 172377776 | 172378274 | 499 | 3' UTR | Pigm | phosphatidylinositol glycan anchor biosynthesis, class M |
| Chr1 | 45391912 | 45393074 | 1163 | Exon | Col5a2 | collagen, type V, alpha 2 |
| Chr1 | 66831388 | 66838302 | 6915 | 3' UTR | Acadl | acyl-coenzyme A dehydrogenase, long-chain |
| Chr1 | 72861984 | 72862232 | 249 | 3' UTR | Igfbp5 | insulin-like growth factor-binding protein 5 |
| Chr1 | 72862380 | 72863213 | 834 | Exon | Igfbp5 | insulin-like growth factor-binding protein 5 |
| Chr1 | 119416702 | 119418087 | 1386 | 3' UTR | Inhbb | inhibin beta-B |
| Chr1 | 119421753 | 119422199 | 447 | 5' UTR | Inhbb | inhibin beta-B |
| Chr1 | 155275856 | 155276054 | 199 | 3' UTR | Xpr1 | xenotropic and polytropic retrovirus receptor 1 |
| Chr1 | 155276651 | 155276949 | 299 | 3' UTR | Xpr1 | xenotropic and polytropic retrovirus receptor 1 |
| Chr1 | 155280376 | 155280923 | 548 | 3' UTR | Xpr1 | xenotropic and polytropic retrovirus receptor 1 |
| Chr1 | 156653766 | 156654458 | 693 | 3' UTR | Tor3a | torsin family 3, member A |
| Chr1 | 171286525 | 171286823 | 299 | Exon | Usp21 | Ubiquitin-specific peptidase 21 |
| Chr1 | 172210140 | 172210288 | 149 | 3' UTR | Casq1 | calsequestrin 1 |
| Chr1 | 172630964 | 172631794 | 831 | 3' UTR | Dusp23 | dual-specificity phosphatase 23 |
| Chr1 | 174015059 | 174026553 | 11495 | Exon | Ifi205 | interferon activated gene 205 |
| Chr10 | 13008499 | 13009134 | 636 | 5' UTR | Sf3b5 | splicing factor 3b, subunit 5 |
| Chr10 | 34297521 | 34298821 | 1301 | 3' UTR | Tspyl4 | TSPY-like 4 |
| Chr10 | 52438208 | 52439051 | 844 | 3' UTR | Nus1 | NUS1 dehydrodolichyl diphosphate synthase subunit |
| Chr10 | 77644504 | 77645346 | 843 | 3' UTR | Ube2g2 | ubiquitin-conjugating enzyme E2G 2 |
| Chr10 | 80174257 | 80174980 | 724 | 3' UTR | 1600002K03Rik | RIKEN cDNA 1600002K03 gene |
| Chr10 | 81387280 | 81388203 | 924 | 3' UTR | Dohh | deoxyhypusine hydroxylase/monooxygenase |
| Chr10 | 83364386 | 83366167 | 1782 | 3' UTR | D10Wsu102e | DNA segment, Chr 10, Wayne State University 102, expressed |
| Chr10 | 85117399 | 85117548 | 150 | 3' UTR | Tmem263 | transmembrane protein 263 |
| Chr10 | 96617100 | 96617299 | 200 | 5' UTR | Btg1 | B cell translocation gene 1, anti-proliferative |
| Chr10 | 37135684 | 37135834 | 151 | 3' UTR | Marcks | myristoylated alanine-rich protein kinase C substrate |
| Chr10 | 40287951 | 40288247 | 297 | 3' UTR | Amd1 | S-adenosylmethionine decarboxylase 1 |
| Chr10 | 80036097 | 80037252 | 1156 | 3' UTR | Polr2e | polymerase (RNA) II (DNA directed) polypeptide E |
| Chr10 | 80900184 | 80900773 | 590 | 5' UTR | Timm13 | translocase of inner mitochondrial membrane 13 |
| Chr10 | 93583459 | 93588004 | 4546 | 3' UTR | Ccdc38 | coiled-coil domain containing 38 |
| Chr10 | 127056100 | 127056599 | 500 | 3' UTR | March9 | membrane-associated ring finger (C3HC4) 9 |
| Chr10 | 128210791 | 128211745 | 955 | 3' UTR | Spryd4 | SPRY domain containing 4 |
| Chr10 | 128548210 | 128548409 | 200 | 3' UTR | Rpl41 | ribosomal protein L41 |
| Chr11 | 22972105 | 22973103 | 999 | 5' UTR | Commd1 | COMM domain containing 1 |
| Chr11 | 22973302 | 22974750 | 1449 | 3' UTR | Commd1 | COMM domain containing 1 |
| Chr11 | 59948362 | 59961035 | 12674 | 3' UTR | Med9 | mediator complex subunit 9 |
| Chr11 | 60443077 | 60443327 | 251 | 3' UTR | Gid4 | GID complex subunit 4, VID24 homolog |
| Chr11 | 60538081 | 60538380 | 300 | 5' UTR | Alkbh5 | alkB homolog 5, RNA demethylase |
| Chr11 | 67798320 | 67798731 | 412 | 5' UTR | Dhrs7c | dehydrogenase/reductase (SDR family) member 7C |
| Chr11 | 69059872 | 69061138 | 1267 | 3' UTR | Borcs6 | BLOC-1-related complex subunit 6 |
| Chr11 | 76986948 | 76987291 | 344 | 3' UTR | Blmh | bleomycin hydrolase |
| Chr11 | 77518679 | 77519179 | 501 | 3' UTR | Abhd15 | abhydrolase domain containing 15 |
| Chr11 | 78539979 | 78540970 | 992 | Exon | Ift20 | intraflagellar transport 20 |
| Chr11 | 82057978 | 82062516 | 4539 | 3' UTR | Ccl11 | chemokine (C-C motif) ligand 11 |
| Chr11 | 83956012 | 83962346 | 6335 | 3' UTR | Ddx52 | DEAD (Asp-Glu-Ala-Asp) box polypeptide 52 |
| Chr11 | 85835926 | 85838428 | 2503 | Exon | Tbx2 | T-box 2 |
| Chr11 | 85840909 | 85841157 | 249 | 3' UTR | Tbx2 | T-box 2 |
| Chr11 | 87116160 | 87122275 | 6116 | 3' UTR | Ska2 | spindle- and kinetochore-associated complex subunit 2 |
| Chr11 | 94211553 | 94211703 | 151 | 5' UTR | Tob1 | transducer of ErbB-2.1 |
| Chr11 | 94214700 | 94214898 | 199 | 3' UTR | Tob1 | transducer of ErbB-2.1 |
| Chr11 | 94641232 | 94643330 | 2099 | Exon | Lrrc59 | leucine-rich repeat containing 59 |
| Chr11 | 94952095 | 94952245 | 151 | 3' UTR | Col1a1 | collagen, type I, alpha 1 |
| Chr11 | 106262218 | 106263112 | 895 | 3' UTR | Psmc5 | protease (prosome, macropain) 26S subunit, ATPase 5 |
| Chr11 | 111074926 | 111075526 | 601 | 3' UTR | Kcnj2 | potassium inwardly rectifying channel, subfamily J, member 2 |
| Chr11 | 120498462 | 120499062 | 601 | 3' UTR | Slc25a10 | solute carrier family 25 (mitochondrial carrier, dicarboxylate transporter), member 10 |
| Chr11 | 3907121 | 3907663 | 543 | 3' UTR | Slc35e4 | solute carrier family 35, member E4 |
| Chr11 | 3912790 | 3913530 | 741 | 5' UTR | Slc35e4 | solute carrier family 35, member E4 |
| Chr11 | 5801732 | 5803082 | 1351 | Exon | Pgam2 | phosphoglycerate mutase 2 |
| Chr11 | 35809763 | 35816642 | 6880 | Exon | Rars | arginyl-tRNA synthetase |
| Chr11 | 49180674 | 49187040 | 6367 | 5' UTR | Btnl9 | butyrophilin-like 9 |
| Chr11 | 53261610 | 53268513 | 6904 | 3' UTR | Hspa4 | heat shock protein 4 |
| Chr11 | 53655605 | 53674967 | 19363 | Exon | Rad50 | RAD50 double-strand break repair protein |
| Chr11 | 53694977 | 53695391 | 415 | Exon | Rad50 | RAD50 double-strand break repair protein |
| Chr11 | 60905167 | 60905810 | 644 | 3' UTR | Natd1 | N-acetyltransferase domain containing 1 |
| Chr11 | 62267365 | 62267506 | 142 | 3' UTR | Zswim7 | zinc finger SWIM-type containing 7 |
| Chr11 | 69734246 | 69735544 | 1299 | 3' UTR | Polr2a | polymerase (RNA) II (DNA directed) polypeptide A |
| Chr11 | 69879056 | 69880094 | 1039 | 3' UTR | Kctd11 | potassium channel tetramerisation domain containing 11 |
| Chr11 | 70395016 | 70395374 | 359 | Exon | Pelp1 | proline, glutamic acid, and leucine-rich protein 1 |
| Chr11 | 70652895 | 70654650 | 1756 | 5' UTR | Pfn1 | profilin 1 |
| Chr11 | 73137357 | 73137505 | 149 | Exon | Gsg2 | germ cell-specific gene 2 |
| Chr11 | 79516217 | 79523613 | 7397 | 5' UTR | Evi2b | ecotropic viral integration site 2b |
| Chr11 | 79516209 | 79516654 | 446 | Exon | Evi2b | ecotropic viral integration site 2b |
| Chr11 | 101278018 | 101278852 | 835 | 3' UTR | Coa3 | cytochrome C oxidase assembly factor 3 |
| Chr11 | 103730342 | 103730590 | 249 | 3' UTR | Wnt9b | wingless-type MMTV integration site family, member 9B |
| Chr11 | 106249193 | 106251255 | 2063 | 3' UTR | Ftsj3 | FtsJ RNA methyltransferase homolog 3 (E. coli) |
| Chr11 | 119288460 | 119288707 | 248 | 3' UTR | Eif4a3 | eukaryotic translation initiation factor 4A3 |
| Chr11 | 119343588 | 119345029 | 1442 | 3' UTR | Card14 | caspase recruitment domain family, member 14 |
| Chr11 | 120807106 | 120807406 | 301 | 3' UTR | Fasn | fatty acid synthase |
| Chr12 | 3365682 | 3366132 | 451 | 5' UTR | Kif3c | kinesin family member 3C |
| Chr12 | 4234176 | 4236656 | 2481 | 5' UTR | Cenpo | centromere protein O |
| Chr12 | 32379580 | 32380619 | 1040 | 3' UTR | Ccdc71l | coiled-coil domain containing 71 like |
| Chr12 | 59067018 | 59072134 | 5117 | Exon | Pnn | pinin |
| Chr12 | 59072233 | 59072828 | 596 | 3' UTR | Pnn | pinin |
| Chr12 | 84017378 | 84017573 | 196 | 3' UTR | Acot1 | acyl-CoA thioesterase 1 |
| Chr12 | 84109045 | 84109291 | 247 | Exon | Acot6 | acyl-CoA thioesterase 6 |
| Chr12 | 98275275 | 98276472 | 1198 | 3' UTR | Gpr65 | G protein-coupled receptor 65 |
| Chr12 | 100113089 | 100115425 | 2337 | Exon | Psmc1 | protease (prosome, macropain) 26S subunit, ATPase 1 |
| Chr12 | 105685451 | 105700869 | 15419 | 3' UTR | Gskip | GSK3B interacting protein |
| Chr12 | 113152111 | 113153780 | 1670 | 5' UTR | Crip1 | cysteine-rich protein 1 (intestinal) |
| Chr12 | 25094141 | 25095315 | 1175 | 3' UTR | Id2 | inhibitor of DNA binding 2 |
| Chr12 | 25095701 | 25095994 | 294 | Exon | Id2 | inhibitor of DNA binding 2 |
| Chr12 | 31331661 | 31333948 | 2288 | 3' UTR | Dld | dihydrolipoamide dehydrogenase |
| Chr12 | 31708761 | 31711692 | 2932 | 5' UTR | Cog5 | component of oligomeric golgi complex 5 |
| Chr12 | 31711792 | 31713776 | 1985 | 5' UTR | Cog5 | component of oligomeric golgi complex 5 |
| Chr12 | 55489557 | 55489754 | 198 | 3' UTR | Nfkbia | nuclear factor of kappa light polypeptide gene enhancer in B cells inhibitor, alpha |
| Chr12 | 76587557 | 76598634 | 11078 | Exon | Sptb | spectrin beta, erythrocytic |
| Chr12 | 76606508 | 76609076 | 2569 | Exon | Sptb | spectrin beta, erythrocytic |
| Chr12 | 78847788 | 78848496 | 709 | Exon | Atp6v1d | ATPase, H^+^ transporting, lysosomal V1 subunit D |
| Chr12 | 80107859 | 80108207 | 349 | 3' UTR | Zfp36l1 | zinc finger protein 36, C3H type-like 1 |
| Chr12 | 86069878 | 86078046 | 8169 | 5' UTR | Tgfb3 | transforming growth factor, beta 3 |
| Chr12 | 86883279 | 86883526 | 248 | Exon | Irf2bpl | interferon regulatory factor 2-binding protein-like |
| Chr12 | 86883625 | 86884764 | 1140 | 5' UTR | Irf2bpl | interferon regulatory factor 2-binding protein-like |
| Chr12 | 90727087 | 90727585 | 499 | 3' UTR | Dio2 | deiodinase, iodothyronine, type II |
| Chr12 | 102753614 | 102757713 | 4100 | 3' UTR | AK010878 | cDNA sequence AK010878 |
| Chr12 | 112772489 | 112773786 | 1298 | 3' UTR | Ahnak2 | AHNAK nucleoprotein 2 |
| Chr13 | 13437702 | 13437902 | 201 | Exon | Nid1 | nidogen 1 |
| Chr13 | 32802279 | 32802478 | 200 | Exon | Mylk4 | myosin light chain kinase family, member 4 |
| Chr13 | 32821098 | 32822361 | 1264 | 3' UTR | Wrnip1 | Werner helicase-interacting protein 1 |
| Chr13 | 42305289 | 42307145 | 1857 | 3' UTR | Edn1 | endothelin 1 |
| Chr13 | 49514848 | 49522782 | 7935 | Exon | Cenpp | centromere protein P |
| Chr13 | 64184641 | 64185303 | 663 | Exon | Habp4 | hyaluronic acid-binding protein 4 |
| Chr13 | 67181753 | 67182900 | 1148 | Exon | Rsl1 | regulator of sex-limited protein 1 |
| Chr13 | 73331846 | 73331988 | 143 | 3' UTR | Mrpl36 | mitochondrial ribosomal protein L36 |
| Chr13 | 93940523 | 93940823 | 301 | 3' UTR | Arsb | arylsulfatase B |
| Chr13 | 94788989 | 94842671 | 53683 | Exon | Tbca | tubulin cofactor A |
| Chr13 | 97245095 | 97250594 | 5500 | 3' UTR | Enc1 | ectodermal-neural cortex 1 |
| Chr13 | 99228720 | 99229212 | 493 | Exon | Zfp366 | zinc finger protein 366 |
| Chr13 | 99234195 | 99246983 | 12789 | 3' UTR | Zfp366 | zinc finger protein 366 |
| Chr13 | 112506464 | 112506612 | 149 | 3' UTR | Il6st | interleukin 6 signal transducer |
| Chr13 | 14610349 | 14610684 | 336 | 3' UTR | Mrpl32 | mitochondrial ribosomal protein L32 |
| Chr13 | 24818053 | 24820723 | 2671 | Exon | Acot13 | acyl-CoA thioesterase 13 |
| Chr13 | 58127168 | 58127664 | 497 | 3' UTR | Hnrnpa0 | heterogeneous nuclear ribonucleoprotein A0 |
| Chr13 | 58127812 | 58128259 | 448 | Exon | Hnrnpa0 | heterogeneous nuclear ribonucleoprotein A0 |
| Chr13 | 58381531 | 58384267 | 2737 | 3' UTR | 2210016F16Rik | RIKEN cDNA 2210016F16 gene |
| Chr13 | 60176790 | 60177535 | 746 | 5' UTR | Gas1 | growth arrest-specific 1 |
| Chr13 | 67041620 | 67041819 | 200 | Exon | Zfp712 | zinc finger protein 712 |
| Chr13 | 67343151 | 67343350 | 200 | Exon | Zfp953 | zinc finger protein 953 |
| Chr13 | 67389773 | 67390616 | 844 | 3' UTR | Zfp429 | zinc finger protein 429 |
| Chr13 | 67490364 | 67491641 | 1278 | 3' UTR | Zfp58 | zinc finger protein 58 |
| Chr13 | 67540129 | 67540427 | 299 | 3' UTR | Zfp748 | zinc finger protein 748 |
| Chr13 | 67592230 | 67592628 | 399 | Exon | Zfp729b | zinc finger protein 729b |
| Chr13 | 67592778 | 67593077 | 300 | Exon | Zfp729b | zinc finger protein 729b |
| Chr13 | 93498511 | 93499209 | 699 | Exon | Jmy | junction-mediating and regulatory protein |
| Chr13 | 95512558 | 95513200 | 643 | 3' UTR | F2rl1 | coagulation factor II (thrombin) receptor-like 1 |
| Chr13 | 95602688 | 95604435 | 1748 | 3' UTR | F2r | coagulation factor II (thrombin) receptor |
| Chr13 | 95604735 | 95618383 | 13649 | 5' UTR | F2r | coagulation factor II (thrombin) receptor |
| Chr13 | 112918591 | 112927331 | 8741 | Exon | Skiv2l2 | superkiller viralicidic activity 2-like 2 (S. cerevisiae) |
| Chr13 | 115101715 | 115101864 | 150 | 5' UTR | Itga1 | integrin alpha 1 |
| Chr13 | 115088404 | 115090109 | 1706 | 5' UTR | Itga1 | integrin alpha 1 |
| Chr14 | 19768356 | 19768904 | 549 | Exon | Nid2 | nidogen 2 |
| Chr14 | 34403938 | 34404287 | 350 | 3' UTR | Mmrn2 | multimerin 2 |
| Chr14 | 52294122 | 52294271 | 150 | 3' UTR | Tox4 | TOX high-mobility group box family member 4 |
| Chr14 | 54368974 | 54369123 | 150 | 3' UTR | Oxa1l | oxidase assembly 1-like |
| Chr14 | 55871204 | 55872050 | 847 | Exon | Nynrin | NYN domain and retroviral integrase containing |
| Chr14 | 69040702 | 69041002 | 301 | 3' UTR | Stc1 | stanniocalcin 1 |
| Chr14 | 69782276 | 69782524 | 249 | Exon | Tnfrsf10b | tumor necrosis factor receptor superfamily, member 10b |
| Chr14 | 120503617 | 120504064 | 448 | 3' UTR | Rap2a | Ras-related protein 2a |
| Chr14 | 18204206 | 18204405 | 200 | 3' UTR | Nr1d2 | nuclear receptor subfamily 1, group D, member 2 |
| Chr14 | 21984507 | 21984656 | 150 | 3' UTR | Zfp503 | zinc finger protein 503 |
| Chr14 | 30999970 | 31000161 | 192 | 3' UTR | Spcs1 | signal peptidase complex subunit 1 homolog (S. cerevisiae) |
| Chr14 | 34370857 | 34374620 | 3764 | 5' UTR | Sncg | synuclein, gamma |
| Chr14 | 50915764 | 50916163 | 400 | 3' UTR | Osgep | O-sialoglycoprotein endopeptidase |
| Chr14 | 52160569 | 52160718 | 150 | 3' UTR | Rpgrip1 | retinitis pigmentosa GTPase regulator interacting protein 1 |
| Chr14 | 52176662 | 52178345 | 1684 | Exon | Supt16 | suppressor of Ty 16 |
| Chr14 | 54568850 | 54569516 | 667 | 3' UTR | Ajuba | ajuba LIM protein |
| Chr14 | 54576428 | 54577464 | 1037 | 5' UTR | Ajuba | ajuba LIM protein |
| Chr14 | 61603135 | 61614191 | 11057 | 5' UTR | Kcnrg | potassium channel regulator |
| Chr14 | 63373183 | 63374222 | 1040 | 3' UTR | Blk | B lymphoid kinase |
| Chr14 | 102978914 | 102979162 | 249 | 3' UTR | Kctd12 | potassium channel tetramerisation domain containing 12 |
| Chr15 | 79094201 | 79094350 | 150 | 3' UTR | Eif3l | eukaryotic translation initiation factor 3, subunit L |
| Chr15 | 79151356 | 79151679 | 324 | Exon | Polr2f | polymerase (RNA) II (DNA directed) polypeptide F |
| Chr15 | 84935344 | 84938395 | 3052 | Exon | Nup50 | nucleoporin 50 |
| Chr15 | 88862921 | 88865035 | 2115 | 3' UTR | Pim3 | proviral integration site 3 |
| Chr15 | 95094409 | 95094906 | 498 | Exon | Tmem117 | transmembrane protein 117 |
| Chr15 | 96370678 | 96370977 | 300 | Exon | Arid2 | AT-rich interactive domain 2 (ARID, RFX-like) |
| Chr15 | 102334337 | 102337169 | 2833 | Exon | Myg1 | melanocyte proliferating gene 1 |
| Chr15 | 3965561 | 3965711 | 151 | 3' UTR | Fbxo4 | F-box protein 4 |
| Chr15 | 34484121 | 34484370 | 250 | 3' UTR | Rida | reactive intermediate imine deaminase A homolog |
| Chr15 | 35932022 | 35937223 | 5202 | 3' UTR | Cox6c | cytochrome c oxidase subunit VIc |
| Chr15 | 53345639 | 53345936 | 298 | 5' UTR | Ext1 | exostoses (multiple) 1 |
| Chr15 | 54252200 | 54252349 | 150 | Exon | Tnfrsf11b | tumor necrosis factor receptor superfamily, member 11b (osteoprotegerin) |
| Chr15 | 77762967 | 77763263 | 297 | Exon | Myh9 | myosin, heavy polypeptide 9, non-muscle |
| Chr15 | 79675996 | 79677263 | 1268 | 3' UTR | Josd1 | Josephin domain containing 1 |
| Chr15 | 81365093 | 81365240 | 148 | 3' UTR | St13 | suppression of tumorigenicity 13 |
| Chr15 | 89371736 | 89373818 | 2083 | 5' UTR | Sco2 | SCO2 cytochrome c oxidase assembly protein |
| Chr15 | 89371981 | 89373969 | 1989 | 5' UTR | Sco2 | SCO2 cytochrome c oxidase assembly protein |
| Chr15 | 89416703 | 89416853 | 151 | 3' UTR | Cpt1b | carnitine palmitoyltransferase 1b, muscle |
| Chr15 | 89472626 | 89473124 | 499 | 3' UTR | Arsa | arylsulfatase A |
| Chr16 | 14706812 | 14708403 | 1592 | 3' UTR | Snai2 | snail family zinc finger 2 |
| Chr16 | 18776896 | 18778213 | 1318 | 5' UTR | Cldn5 | claudin 5 |
| Chr16 | 18836764 | 18840113 | 3350 | 3' UTR | 2510002D24Rik | RIKEN cDNA 2510002D24 gene |
| Chr16 | 35155686 | 35156182 | 497 | 5' UTR | Adcy5 | adenylate cyclase 5 |
| Chr16 | 37878376 | 37881964 | 3589 | 3' UTR | Lrrc58 | leucine-rich repeat containing 58 |
| Chr16 | 37885960 | 37886110 | 151 | 3' UTR | Lrrc58 | leucine-rich repeat containing 58 |
| Chr16 | 90749330 | 90749726 | 397 | 3' UTR | Mrap | melanocortin 2 receptor accessory protein |
| Chr16 | 92111984 | 92112178 | 195 | 3' UTR | Mrps6 | mitochondrial ribosomal protein S6 |
| Chr16 | 96493827 | 96525642 | 31816 | 3' UTR | Pcp4 | Purkinje cell protein 4 |
| Chr16 | 5087337 | 5089429 | 2093 | Exon | Ppl | periplakin |
| Chr16 | 5089529 | 5092370 | 2842 | Exon | Ppl | periplakin |
| Chr16 | 17130282 | 17131716 | 1435 | 3' UTR | Sdf2l1 | stromal cell-derived factor 2-like 1 |
| Chr16 | 18622503 | 18622918 | 416 | 3' UTR | Sept5 | septin 5 |
| Chr16 | 30269848 | 30270889 | 1042 | 3' UTR | Lrrc15 | leucine-rich repeat containing 15 |
| Chr16 | 38598393 | 38598642 | 250 | 3' UTR | Arhgap31 | Rho GTPase activating protein 31 |
| Chr16 | 57599440 | 57599937 | 498 | Exon | Cmss1 | cms small ribosomal subunit 1 |
| Chr16 | 85802366 | 85802515 | 150 | Exon | Adamts1 | a disintegrin-like and metallopeptidase (reprolysin type) with thrombospondin type 1 motif, 1 |
| Chr17 | 6979005 | 6988745 | 9741 | Exon | Rnaset2b | ribonuclease T2B |
| Chr17 | 15728221 | 15730817 | 2597 | Exon | Chd1 | chromodomain helicase DNA-binding protein 1 |
| Chr17 | 15766390 | 15770869 | 4480 | 3' UTR | Chd1 | chromodomain helicase DNA-binding protein 1 |
| Chr17 | 17264268 | 17264563 | 296 | Exon | LOC102640673 | zinc finger protein 431-like |
| Chr17 | 17264661 | 17265252 | 592 | Exon | LOC102640673 | zinc finger protein 431-like |
| Chr17 | 17265350 | 17265990 | 641 | Exon | LOC102640673 | zinc finger protein 431-like |
| Chr17 | 17318166 | 17318900 | 735 | Exon | Gm6712 | predicted gene 6712 |
| Chr17 | 24470769 | 24471115 | 347 | Exon | Pgp | phosphoglycolate phosphatase |
| Chr17 | 25823727 | 25823877 | 151 | 5' UTR | Wdr24 | WD repeat domain 24 |
| Chr17 | 25830748 | 25830997 | 250 | 3' UTR | Jmjd8 | jumonji domain containing 8 |
| Chr17 | 26126274 | 26126468 | 195 | 3' UTR | Mrpl28 | mitochondrial ribosomal protein L28 |
| Chr17 | 28220193 | 28228509 | 8317 | 3' UTR | Def6 | differentially expressed in FDCP 6 |
| Chr17 | 29491143 | 29491814 | 672 | 5' UTR | Pim1 | proviral integration site 1 |
| Chr17 | 29704333 | 29704582 | 250 | 3' UTR | Ccdc167 | coiled-coil domain containing 167 |
| Chr17 | 33301852 | 33303096 | 1245 | Exon | Zfp955b | zinc finger protein 955B |
| Chr17 | 34204579 | 34205640 | 1062 | 5' UTR | Tap2 | transporter 2, ATP-binding cassette, subfamily B (MDR/TAP) |
| Chr17 | 34590930 | 34591704 | 775 | 3' UTR | Gpsm3 | G protein-signaling modulator 3 (AGS3-like, C. elegans) |
| Chr17 | 35263385 | 35265839 | 2455 | Exon | H2-D1 | histocompatibility 2, D region locus 1 |
| Chr17 | 35424926 | 35427989 | 3064 | Exon | H2-Q6 | histocompatibility 2, Q region locus 6 |
| Chr17 | 35428087 | 35428361 | 275 | Exon | H2-Q6 | histocompatibility 2, Q region locus 6 |
| Chr17 | 43017002 | 43039846 | 22845 | Exon | Tnfrsf21 | tumor necrosis factor receptor superfamily, member 21 |
| Chr17 | 56047376 | 56059058 | 11683 | Exon | Chaf1a | chromatin assembly factor 1, subunit A (p150) |
| Chr17 | 56065008 | 56067878 | 2871 | 3' UTR | Chaf1a | chromatin assembly factor 1, subunit A (p150) |
| Chr17 | 56171739 | 56173019 | 1281 | 3' UTR | Tnfaip8l1 | tumor necrosis factor, alpha-induced protein 8-like 1 |
| Chr17 | 12420120 | 12421254 | 1135 | 3' UTR | Slc22a3 | solute carrier family 22 (organic cation transporter), member 3 |
| Chr17 | 14666595 | 14666993 | 399 | 3' UTR | Thbs2 | thrombospondin 2 |
| Chr17 | 21929096 | 21929294 | 199 | Exon | Zfp942 | zinc finger protein 942 |
| Chr17 | 22201347 | 22202761 | 1415 | Exon | Gm4944 | predicted gene 4944 |
| Chr17 | 24048702 | 24055968 | 7267 | 3' UTR | Kctd5 | potassium channel tetramerisation domain containing 5 |
| Chr17 | 25830734 | 25830882 | 149 | 3' UTR | Jmjd8 | jumonji domain containing 8 |
| Chr17 | 27144363 | 27144657 | 295 | 3' UTR | Ip6k3 | inositol hexaphosphate kinase 3 |
| Chr17 | 28400595 | 28401093 | 499 | 3' UTR | Fkbp5 | FK506-binding protein 5 |
| Chr17 | 28402649 | 28402848 | 200 | Exon | Fkbp5 | FK506-binding protein 5 |
| Chr17 | 31845241 | 31845439 | 199 | 3' UTR | Sik1 | salt-inducible kinase 1 |
| Chr17 | 34957267 | 34957809 | 543 | Exon | Hspa1b | heat shock protein 1B |
| Chr17 | 56269561 | 56270008 | 448 | 3' UTR | Ticam1 | toll-like receptor adaptor molecule 1 |
| Chr17 | 56270107 | 56271149 | 1043 | Exon | Ticam1 | toll-like receptor adaptor molecule 1 |
| Chr17 | 56608919 | 56609677 | 759 | 5' UTR | 2410015M20Rik | RIKEN cDNA 2410015M20 gene |
| Chr17 | 57022448 | 57022948 | 501 | 3' UTR | Khsrp | KH-type splicing regulatory protein |
| Chr17 | 80397887 | 80398585 | 699 | 3' UTR | Arhgef33 | Rho guanine nucleotide exchange factor (GEF) 33 |
| Chr17 | 80433542 | 80433692 | 151 | Exon | Sos1 | son of sevenless homolog 1 (Drosophila) |
| Chr17 | 84187012 | 84187648 | 637 | Exon | Zfp36l2 | zinc finger protein 36, C3H type-like 2 |
| Chr18 | 10617894 | 10628033 | 10140 | 5' UTR | Snrpd1 | small nuclear ribonucleoprotein D1 |
| Chr18 | 20598350 | 20601790 | 3441 | Exon | Dsg2 | desmoglein 2 |
| Chr18 | 20603233 | 20603531 | 299 | 3' UTR | Dsg2 | desmoglein 2 |
| Chr18 | 34861603 | 34864114 | 2512 | 3' UTR | Egr1 | early growth response 1 |
| Chr18 | 35572105 | 35572652 | 548 | Exon | Matr3 | matrin 3 |
| Chr18 | 36647149 | 36647348 | 200 | Exon | Ankhd1 | ankyrin repeat and KH domain containing 1 |
| Chr18 | 36647448 | 36647648 | 201 | Exon | Ankhd1 | ankyrin repeat and KH domain containing 1 |
| Chr18 | 36647797 | 36648396 | 600 | Exon | Ankhd1 | ankyrin repeat and KH domain containing 1 |
| Chr18 | 36744756 | 36756614 | 11859 | 5' UTR | Ik | IK cytokine |
| Chr18 | 37505413 | 37506711 | 1299 | Exon | Gm37013 | predicted gene, 37013 |
| Chr18 | 37686297 | 37687393 | 1097 | Exon | Gm37013 | predicted gene, 37013 |
| Chr18 | 37716172 | 37716420 | 249 | Exon | Gm37013 | predicted gene, 37013 |
| Chr18 | 37806510 | 37807507 | 998 | 5' UTR | Gm37013 | predicted gene, 37013 |
| Chr18 | 37808005 | 37808803 | 799 | Exon | Gm37013 | predicted gene, 37013 |
| Chr18 | 62950163 | 62952695 | 2533 | 3' UTR | Apcdd1 | adenomatosis polyposis coli down-regulated 1 |
| Chr18 | 67390781 | 67393002 | 2222 | Exon | Tubb6 | tubulin, beta 6 class V |
| Chr18 | 6216795 | 6220929 | 4135 | Exon | Kif5b | kinesin family member 5B |
| Chr18 | 34938062 | 34939009 | 948 | 3' UTR | Hspa9 | heat shock protein 9 |
| Chr18 | 36725210 | 36726620 | 1411 | 5' UTR | Cd14 | CD14 antigen |
| Chr18 | 36742563 | 36744270 | 1708 | 3' UTR | Ndufa2 | NADH dehydrogenase (ubiquinone) 1 alpha subcomplex, 2 |
| Chr18 | 37642166 | 37644105 | 1940 | 5' UTR | Gm37013 | predicted gene, 37013 |
| Chr18 | 40218789 | 40219300 | 512 | 5' UTR | Yipf5 | Yip1 domain family, member 5 |
| Chr18 | 46559813 | 46559961 | 149 | 3' UTR | Ticam2 | toll-like receptor adaptor molecule 2 |
| Chr19 | 5069605 | 5070640 | 1036 | 3' UTR | Cd248 | CD248 antigen, endosialin |
| Chr19 | 6061595 | 6062420 | 826 | Exon | Znhit2 | zinc finger, HIT domain containing 2 |
| Chr19 | 6090380 | 6090650 | 271 | Exon | Cdca5 | cell division cycle-associated 5 |
| Chr19 | 8771096 | 8771295 | 200 | Exon | Tmem223 | transmembrane protein 223 |
| Chr19 | 8889145 | 8890659 | 1515 | 3' UTR | 1810009A15Rik | RIKEN cDNA 1810009A15 gene |
| Chr19 | 8925849 | 8926279 | 431 | Exon | B3gat3 | beta-1,3-glucuronyltransferase 3 (glucuronosyltransferase I) |
| Chr19 | 24678332 | 24678480 | 149 | 3' UTR | Pgm5 | phosphoglucomutase 5 |
| Chr19 | 28964367 | 28965261 | 895 | 3' UTR | 4430402I18Rik | RIKEN cDNA 4430402I18 gene |
| Chr19 | 31083088 | 31084914 | 1827 | Exon | Cstf2t | cleavage stimulation factor, 3' pre-RNA subunit 2, tau |
| Chr19 | 32389315 | 32390041 | 727 | 5' UTR | Sgms1 | sphingomyelin synthase 1 |
| Chr19 | 41831055 | 41831498 | 444 | 3' UTR | Frat1 | frequently rearranged in advanced T cell lymphomas |
| Chr19 | 42147885 | 42148282 | 398 | 3' UTR | Marveld1 | MARVEL (membrane-associating) domain containing 1 |
| Chr19 | 53620610 | 53625100 | 4491 | Exon | Smc3 | structural maintenance of chromosomes 3 |
| Chr19 | 56722770 | 56722970 | 201 | Exon | Adrb1 | adrenergic receptor, beta 1 |
| Chr19 | 4453890 | 4456602 | 2713 | Exon | Syt12 | synaptotagmin XII |
| Chr19 | 4456702 | 4476994 | 20293 | 5' UTR | Syt12 | synaptotagmin XII |
| Chr19 | 6068239 | 6068867 | 629 | Exon | Vps51 | VPS51 GARP complex subunit |
| Chr19 | 6136000 | 6141137 | 5138 | 5' UTR | Arl2 | ADP-ribosylation factor-like 2 |
| Chr19 | 8880063 | 8880884 | 822 | 5' UTR | Uqcc3 | ubiquinol-cytochrome c reductase complex assembly factor 3 |
| Chr19 | 10942662 | 10948479 | 5818 | 3' UTR | Ccdc86 | coiled-coil domain containing 86 |
| Chr19 | 24261801 | 24280437 | 18637 | 3' UTR | Fxn | frataxin |
| Chr19 | 43499850 | 43500291 | 442 | 3' UTR | Got1 | glutamic-oxaloacetic transaminase 1, soluble |
| Chr2 | 25263583 | 25264026 | 444 | Exon | Tprn | taperin |
| Chr2 | 25272614 | 25273806 | 1193 | Exon | Anapc2 | anaphase promoting complex subunit 2 |
| Chr2 | 29816038 | 29816335 | 298 | 3' UTR | Slc27a4 | solute carrier family 27 (fatty acid transporter), member 4 |
| Chr2 | 32573045 | 32573523 | 479 | 3' UTR | Dpm2 | dolichol-phosphate (beta-D) mannosyltransferase 2 |
| Chr2 | 91057828 | 91059289 | 1462 | Exon | Psmc3 | proteasome (prosome, macropain) 26S subunit, ATPase 3 |
| Chr2 | 119290209 | 119298305 | 8097 | 3' UTR | Vps18 | VPS18 CORVET/HOPS core subunit |
| Chr2 | 122234888 | 122248841 | 13954 | 5' UTR | Sord | sorbitol dehydrogenase |
| Chr2 | 127258045 | 127258292 | 248 | 3' UTR | Tmem127 | transmembrane protein 127 |
| Chr2 | 130564087 | 130565394 | 1308 | 3' UTR | Mrps26 | mitochondrial ribosomal protein S26 |
| Chr2 | 150830851 | 150831001 | 151 | 3' UTR | Pygb | brain glycogen phosphorylase |
| Chr2 | 162931608 | 162932124 | 517 | 5' UTR | Srsf6 | serine/arginine-rich splicing factor 6 |
| Chr2 | 167541930 | 167542274 | 345 | 3' UTR | Snai1 | snail family zinc finger 1 |
| Chr2 | 172446669 | 172463254 | 16586 | 5' UTR | Gcnt7 | glucosaminyl (N-acetyl) transferase family member 7 |
| Chr2 | 25574210 | 25574774 | 565 | Exon | Phpt1 | phosphohistidine phosphatase 1 |
| Chr2 | 26459650 | 26460199 | 550 | Exon | Notch1 | notch 1 |
| Chr2 | 30285761 | 30286206 | 446 | 5' UTR | Dolk | dolichol kinase |
| Chr2 | 73305910 | 73312492 | 6583 | 5' UTR | Cir1 | corepressor interacting with RBPJ, 1 |
| Chr2 | 80413157 | 80424608 | 11452 | 3' UTR | Frzb | frizzled-related protein |
| Chr2 | 80446654 | 80447149 | 496 | 5' UTR | Frzb | frizzled-related protein |
| Chr2 | 84723468 | 84724210 | 743 | 3' UTR | Clp1 | CLP1, cleavage and polyadenylation factor I subunit |
| Chr2 | 91982377 | 91982525 | 149 | 3' UTR | Creb3l1 | cAMP responsive element-binding protein 3-like 1 |
| Chr2 | 93955860 | 93956653 | 794 | 3' UTR | Gm13889 | predicted gene 13889 |
| Chr2 | 113833628 | 113834025 | 398 | Exon | Arhgap11a | Rho GTPase activating protein 11A |
| Chr2 | 118475991 | 118476855 | 865 | 3' UTR | Srp14 | signal recognition particle 14 |
| Chr2 | 121461914 | 121462113 | 200 | 3' UTR | Mfap1b | microfibrillar-associated protein 1B |
| Chr2 | 121467080 | 121469960 | 2881 | Exon | Mfap1b | microfibrillar-associated protein 1B |
| Chr2 | 131171737 | 131172743 | 1007 | Exon | Spef1 | sperm flagellar 1 |
| Chr2 | 131174613 | 131174810 | 198 | 5' UTR | Spef1 | sperm flagellar 1 |
| Chr2 | 144059724 | 144068702 | 8979 | 5' UTR | Banf2 | barrier to autointegration factor 2 |
| Chr2 | 148438847 | 148438996 | 150 | 3' UTR | Cd93 | CD93 antigen |
| Chr2 | 152337474 | 152338596 | 1123 | 3' UTR | Trib3 | tribbles pseudokinase 3 |
| Chr2 | 152931947 | 152932868 | 922 | 3' UTR | Foxs1 | forkhead box S1 |
| Chr2 | 153008939 | 153009086 | 148 | 3' UTR | Pdrg1 | p53 and DNA damage regulated 1 |
| Chr2 | 154884403 | 154892807 | 8405 | 5' UTR | Eif2s2 | eukaryotic translation initiation factor 2, subunit 2 (beta) |
| Chr2 | 158116555 | 158116755 | 201 | 3' UTR | Tgm2 | transglutaminase 2, C polypeptide |
| Chr2 | 164830778 | 164832970 | 2193 | 5' UTR | Ctsa | cathepsin A |
| Chr2 | 168183950 | 168184398 | 449 | Exon | Adnp | activity-dependent neuroprotective protein |
| Chr2 | 168184497 | 168210445 | 25949 | 5' UTR | Adnp | activity-dependent neuroprotective protein |
| Chr3 | 65552691 | 65553540 | 850 | Exon | Tiparp | TCDD-inducible poly(ADP-ribose) polymerase |
| Chr3 | 68494639 | 68616501 | 121863 | 5' UTR | Schip1 | schwannomin interacting protein 1 |
| Chr3 | 87971193 | 87971641 | 449 | 5' UTR | Nes | nestin |
| Chr3 | 87971740 | 87974827 | 3088 | Exon | Nes | nestin |
| Chr3 | 87979903 | 87980152 | 250 | 3' UTR | Nes | nestin |
| Chr3 | 89779768 | 89782606 | 2839 | 3' UTR | Ube2q1 | ubiquitin-conjugating enzyme E2Q family member 1 |
| Chr3 | 93524084 | 93526151 | 2068 | 3' UTR | S100a11 | S100 calcium-binding protein A11 |
| Chr3 | 95871670 | 95887141 | 15472 | 5' UTR | Ciart | circadian-associated repressor of transcription |
| Chr3 | 96185157 | 96185356 | 200 | Exon | Sv2a | synaptic vesicle glycoprotein 2 a |
| Chr3 | 96222652 | 96223294 | 643 | 3' UTR | Hist2h2be | histone cluster 2, H2be |
| Chr3 | 97921337 | 97921534 | 198 | 3' UTR | Sec22b | SEC22 homolog B, vesicle-trafficking protein |
| Chr3 | 98303189 | 98309441 | 6253 | 3' UTR | Hmgcs2 | 3-hydroxy-3-methylglutaryl-coenzyme A synthase 2 |
| Chr3 | 122924494 | 122925609 | 1116 | 3' UTR | 1810037I17Rik | RIKEN cDNA 1810037I17 gene |
| Chr3 | 133102478 | 133109396 | 6919 | Exon | Ints12 | integrator complex subunit 12 |
| Chr3 | 51521132 | 51526937 | 5806 | 3' UTR | Setd7 | SET domain containing (lysine methyltransferase) 7 |
| Chr3 | 58525443 | 58525639 | 197 | 5' UTR | Serp1 | stress-associated endoplasmic reticulum protein 1 |
| Chr3 | 89411211 | 89411813 | 603 | 5' UTR | Flad1 | flavin adenine dinucleotide synthetase 1 |
| Chr3 | 90490255 | 90490915 | 661 | Exon | Snapin | SNAP-associated protein |
| Chr3 | 90512043 | 90514236 | 2194 | 5' UTR | S100a1 | S100 calcium-binding protein A1 |
| Chr3 | 92484003 | 92484299 | 297 | 3' UTR | Sprr1a | small proline-rich protein 1A |
| Chr3 | 106760433 | 106760679 | 247 | 3' UTR | Cd53 | CD53 antigen |
| Chr3 | 123368741 | 123368939 | 199 | 3' UTR | Mettl14 | methyltransferase like 14 |
| Chr3 | 132660644 | 132667433 | 6790 | Exon | Aimp1 | aminoacyl tRNA synthetase complex-interacting multifunctional protein 1 |
| Chr3 | 142793384 | 142794421 | 1038 | 3' UTR | Pkn2 | protein kinase N2 |
| Chr3 | 153922402 | 153922844 | 443 | 3' UTR | Acadm | acyl-coenzyme A dehydrogenase, medium chain |
| Chr4 | 9859361 | 9859759 | 399 | Exon | Gdf6 | growth differentiation factor 6 |
| Chr4 | 9859908 | 9860803 | 896 | 3' UTR | Gdf6 | growth differentiation factor 6 |
| Chr4 | 32983248 | 32995891 | 12644 | 5' UTR | Rragd | Ras-related GTP binding D |
| Chr4 | 41518646 | 41518788 | 143 | 3' UTR | Fam219a | family with sequence similarity 219, member A |
| Chr4 | 57689526 | 57855008 | 165483 | 5' UTR | Akap2 | A kinase (PRKA) anchor protein 2 |
| Chr4 | 66840589 | 66841937 | 1349 | 3' UTR | Tlr4 | toll-like receptor 4 |
| Chr4 | 80910786 | 80953901 | 43116 | 5' UTR | Lurap1l | leucine-rich adaptor protein 1-like |
| Chr4 | 99037851 | 99037997 | 147 | Exon | Angptl3 | angiopoietin-like 3 |
| Chr4 | 99983978 | 99986698 | 2721 | Exon | Pgm2 | phosphoglucomutase 2 |
| Chr4 | 107879854 | 107880949 | 1096 | 5' UTR | Magoh | mago homolog, exon junction complex core component |
| Chr4 | 107884951 | 107887325 | 2375 | 3' UTR | Magoh | mago homolog, exon junction complex core component |
| Chr4 | 108340123 | 108340272 | 150 | 3' UTR | Coa7 | cytochrome c oxidase assembly factor 7 |
| Chr4 | 115791266 | 115797995 | 6730 | Exon | Atpaf1 | ATP synthase mitochondrial F1 complex assembly factor 1 |
| Chr4 | 116096662 | 116096861 | 200 | 3' UTR | Lrrc41 | leucine-rich repeat containing 41 |
| Chr4 | 120666660 | 120667772 | 1113 | 5' UTR | Cited4 | Cbp/p300-interacting transactivator, with Glu/Asp-rich carboxy-terminal domain, 4 |
| Chr4 | 124658830 | 124658978 | 149 | Exon | Pou3f1 | POU domain, class 3, transcription factor 1 |
| Chr4 | 124714961 | 124716463 | 1503 | Exon | Sf3a3 | splicing factor 3a, subunit 3 |
| Chr4 | 129514769 | 129515593 | 825 | 3' UTR | Marcksl1 | MARCKS-like 1 |
| Chr4 | 132535609 | 132553295 | 17687 | 3' UTR | Dnajc8 | DnaJ heat shock protein family (Hsp40) member C8 |
| Chr4 | 133486747 | 133487045 | 299 | Exon | Fam46b | family with sequence similarity 46, member B |
| Chr4 | 135920825 | 135921072 | 248 | Exon | Fuca1 | fucosidase, alpha-L- 1, tissue |
| Chr4 | 135939163 | 135939459 | 297 | 3' UTR | Fuca1 | fucosidase, alpha-L- 1, tissue |
| Chr4 | 146466498 | 146467096 | 599 | Exon | Gm13251 | predicted gene 13251 |
| Chr4 | 147179348 | 147180284 | 937 | 3' UTR | Gm13139 | predicted gene 13139 |
| Chr4 | 147512599 | 147512744 | 146 | Exon | Gm13152 | predicted gene 13152 |
| Chr4 | 150105629 | 150105877 | 249 | 3' UTR | Gpr157 | G protein-coupled receptor 157 |
| Chr4 | 152015780 | 152015930 | 151 | 3' UTR | Klhl21 | kelch-like 21 |
| Chr4 | 42980013 | 42980211 | 199 | 3' UTR | Vcp | valosin containing protein |
| Chr4 | 45423725 | 45424024 | 300 | 3' UTR | Shb | src homology 2 domain-containing transforming protein B |
| Chr4 | 45447635 | 45529781 | 82147 | Exon | Shb | src homology 2 domain-containing transforming protein B |
| Chr4 | 45529980 | 45530529 | 550 | 5' UTR | Shb | src homology 2 domain-containing transforming protein B |
| Chr4 | 49512646 | 49513281 | 636 | 3' UTR | Mrpl50 | mitochondrial ribosomal protein L50 |
| Chr4 | 49536534 | 49536879 | 346 | 3' UTR | Aldob | aldolase B, fructose-bisphosphate |
| Chr4 | 62523844 | 62525014 | 1171 | 5' UTR | Pole3 | polymerase (DNA directed), epsilon 3 (p17 subunit) |
| Chr4 | 94569267 | 94569665 | 399 | 3' UTR | Plaa | phospholipase A2, activating protein |
| Chr4 | 107066517 | 107066816 | 300 | 5' UTR | Mrpl37 | mitochondrial ribosomal protein L37 |
| Chr4 | 108576999 | 108579238 | 2240 | 5' UTR | Orc1 | origin recognition complex, subunit 1 |
| Chr4 | 119530846 | 119531189 | 344 | Exon | Aa415398 | expressed sequence AA415398 |
| Chr4 | 127311470 | 127312956 | 1487 | 3' UTR | Gja4 | gap junction protein, alpha 4 |
| Chr4 | 128774476 | 128777220 | 2745 | 3' UTR | Zfp362 | zinc finger protein 362 |
| Chr4 | 132459173 | 132459863 | 691 | 3' UTR | Med18 | mediator complex subunit 18 |
| Chr4 | 133969156 | 133969304 | 149 | 3' UTR | Dhdds | dehydrodolichyl diphosphate synthase |
| Chr4 | 133970883 | 133971228 | 346 | 3' UTR | Dhdds | dehydrodolichyl diphosphate synthase |
| Chr4 | 134093812 | 134094982 | 1171 | Exon | Cd52 | CD52 antigen |
| Chr4 | 134127735 | 134128659 | 925 | 3' UTR | Sh3bgrl3 | SH3 domain-binding glutamic acid-rich protein-like 3 |
| Chr4 | 136011122 | 136021599 | 10478 | Exon | Tceb3 | transcription elongation factor B (SIII), polypeptide 3 |
| Chr4 | 150897180 | 150905276 | 8097 | 3' UTR | Park7 | Parkinson disease (autosomal recessive, early onset) 7 |
| Chr4 | 155991344 | 155992085 | 742 | 3' UTR | B3galt6 | UDP-Gal:betaGal beta 1,3-galactosyltransferase, polypeptide 6 |
| Chr5 | 3569519 | 3569666 | 148 | 3' UTR | Fam133b | family with sequence similarity 133, member B |
| Chr5 | 20951733 | 20952577 | 845 | 5' UTR | Rsbn1l | round spermatid basic protein 1-like |
| Chr5 | 21372772 | 21375808 | 3037 | Exon | Ccdc146 | coiled-coil domain containing 146 |
| Chr5 | 33027770 | 33027917 | 148 | 3' UTR | Ywhah | tyrosine 3-monooxygenase/tryptophan 5-monooxygenase activation protein, eta polypeptide |
| Chr5 | 52369339 | 52369489 | 151 | 3' UTR | Sod3 | superoxide dismutase 3, extracellular |
| Chr5 | 91517950 | 91594010 | 76061 | Exon | Parm1 | prostate androgen-regulated mucin-like protein 1 |
| Chr5 | 104520988 | 104521736 | 749 | Exon | BC005561 | cDNA sequence BC005561 |
| Chr5 | 111419860 | 111420059 | 200 | Exon | Mn1 | meningioma 1 |
| Chr5 | 111420557 | 111454734 | 34178 | Exon | Mn1 | meningioma 1 |
| Chr5 | 112333159 | 112333409 | 251 | Exon | Tfip11 | tuftelin-interacting protein 11 |
| Chr5 | 123355981 | 123371412 | 15432 | 3' UTR | Bcl7a | B cell CLL/lymphoma 7A |
| Chr5 | 124723756 | 124724006 | 251 | 3' UTR | Atp6v0a2 | ATPase, H+ transporting, lysosomal V0 subunit A2 |
| Chr5 | 124724205 | 124724405 | 201 | 3' UTR | Atp6v0a2 | ATPase, H+ transporting, lysosomal V0 subunit A2 |
| Chr5 | 138191922 | 138193498 | 1577 | 3' UTR | Cnpy4 | canopy FGF signaling regulator 4 |
| Chr5 | 140419651 | 140425284 | 5634 | Exon | Eif3b | eukaryotic translation initiation factor 3, subunit B |
| Chr5 | 140443160 | 140443309 | 150 | 3' UTR | Eif3b | eukaryotic translation initiation factor 3, subunit B |
| Chr5 | 142960602 | 142960750 | 149 | Exon | Fscn1 | fascin actin-bundling protein 1 |
| Chr5 | 23702367 | 23703111 | 745 | 5' UTR | Gm29779 | predicted gene, 29779 |
| Chr5 | 31260676 | 31261019 | 344 | Exon | Ift172 | intraflagellar transport 172 |
| Chr5 | 34177022 | 34177715 | 694 | Exon | Mxd4 | Max dimerization protein 4 |
| Chr5 | 107109546 | 107118455 | 8910 | 3' UTR | Tgfbr3 | transforming growth factor, beta receptor III |
| Chr5 | 114669686 | 114670177 | 492 | 3' UTR | Gltp | glycolipid transfer protein |
| Chr5 | 115333982 | 115341062 | 7081 | 3' UTR | Gatc | glutamyl-tRNA(Gln) amidotransferase, subunit C |
| Chr5 | 124005724 | 124032162 | 26439 | 3' UTR | Vps37b | vacuolar protein sorting 37B |
| Chr5 | 138140747 | 138140946 | 200 | 3' UTR | Zfp113 | zinc finger protein 113 |
| Chr5 | 140327724 | 140331216 | 3493 | 3' UTR | Mrm2 | mitochondrial rRNA methyltransferase 2 |
| Chr5 | 145183849 | 145187180 | 3332 | Exon | Atp5j2 | ATP synthase, H+ transporting, mitochondrial F0 complex, subunit F2 |
| Chr6 | 18848735 | 18851716 | 2982 | Exon | Lsm8 | LSM8 homolog, U6 small nuclear RNA-associated |
| Chr6 | 21723271 | 21727607 | 4337 | 3' UTR | Kcnd2 | potassium voltage-gated channel, Shal-related family, member 2 |
| Chr6 | 28425801 | 28426254 | 454 | 3' UTR | Arf5 | ADP-ribosylation factor 5 |
| Chr6 | 38491254 | 38491404 | 151 | Exon | Ubn2 | ubinuclein 2 |
| Chr6 | 38491504 | 38498709 | 7206 | Exon | Ubn2 | ubinuclein 2 |
| Chr6 | 67036697 | 67063449 | 26753 | 5' UTR | Gadd45a | growth arrest and DNA-damage-inducible 45 alpha |
| Chr6 | 88102115 | 88103608 | 1494 | 3' UTR | Rpn1 | ribophorin I |
| Chr6 | 97233117 | 97233315 | 199 | 3' UTR | Arl6ip5 | ADP-ribosylation factor-like 6 interacting protein 5 |
| Chr6 | 115676099 | 115676296 | 198 | 5' UTR | Raf1 | v-raf-leukemia viral oncogene 1 |
| Chr6 | 115676395 | 115677134 | 740 | 5' UTR | Raf1 | v-raf-leukemia viral oncogene 1 |
| Chr6 | 119480214 | 119481851 | 1638 | 3' UTR | Fbxl14 | F-box and leucine-rich repeat protein 14 |
| Chr6 | 134986488 | 134986786 | 299 | 3' UTR | Apold1 | apolipoprotein L domain containing 1 |
| Chr6 | 136808396 | 136809038 | 643 | 3' UTR | H2afj | H2A histone family, member J |
| Chr6 | 6558471 | 6578511 | 20041 | Exon | Shfm1 | split hand/foot malformation (ectrodactyly) type 1 |
| Chr6 | 14717655 | 14755224 | 37570 | Exon | Ppp1r3a | protein phosphatase 1, regulatory (inhibitor) subunit 3A |
| Chr6 | 29152290 | 29160080 | 7791 | Exon | Rbm28 | RNA-binding motif protein 28 |
| Chr6 | 31528648 | 31528997 | 350 | Exon | Podxl | podocalyxin-like |
| Chr6 | 32148278 | 32148428 | 151 | 3' UTR | Plxna4 | plexin A4 |
| Chr6 | 32148926 | 32149723 | 798 | 3' UTR | Plxna4 | plexin A4 |
| Chr6 | 83053980 | 83054522 | 543 | 5' UTR | Aup1 | ancient ubiquitous protein 1 |
| Chr6 | 89319056 | 89319405 | 350 | 3' UTR | Plxna1 | plexin A1 |
| Chr6 | 99626615 | 99632261 | 5647 | 3' UTR | Eif4e3 | eukaryotic translation initiation factor 4E member 3 |
| Chr6 | 124746910 | 124747459 | 550 | Exon | Atn1 | atrophin 1 |
| Chr6 | 124811222 | 124811545 | 324 | 3' UTR | Tpi1 | triosephosphate isomerase 1 |
| Chr6 | 126639054 | 126639253 | 200 | 3' UTR | Kcna1 | potassium voltage-gated channel, shaker-related subfamily, member 1 |
| Chr6 | 126639353 | 126639552 | 200 | 3' UTR | Kcna1 | potassium voltage-gated channel, shaker-related subfamily, member 1 |
| Chr7 | 16455203 | 16455442 | 240 | 3' UTR | Tmem160 | transmembrane protein 160 |
| Chr7 | 16886954 | 16887152 | 199 | 3' UTR | Dact3 | dishevelled-binding antagonist of beta-catenin 3 |
| Chr7 | 19004165 | 19005964 | 1800 | 5' UTR | Irf2bp1 | interferon regulatory factor 2-binding protein 1 |
| Chr7 | 24530797 | 24531774 | 978 | 5' UTR | Irgq | immunity-related GTPase family, Q |
| Chr7 | 24531874 | 24533572 | 1699 | Exon | Irgq | immunity-related GTPase family, Q |
| Chr7 | 29289416 | 29293342 | 3927 | 3' UTR | Ppp1r14a | protein phosphatase 1, regulatory (inhibitor) subunit 14A |
| Chr7 | 45410184 | 45410534 | 351 | 3' UTR | Kcna7 | potassium voltage-gated channel, shaker-related subfamily, member 7 |
| Chr7 | 81024185 | 81024384 | 200 | Exon | Zfp592 | zinc finger protein 592 |
| Chr7 | 97718397 | 97718647 | 251 | 3' UTR | Clns1a | chloride channel, nucleotide-sensitive, 1A |
| Chr7 | 110627768 | 110629671 | 1904 | 5' UTR | Adm | adrenomedullin |
| Chr7 | 111055827 | 111056327 | 501 | 3' UTR | Ctr9 | CTR9 homolog, Paf1/RNA polymerase II complex component |
| Chr7 | 127541839 | 127541988 | 150 | Exon | Srcap | Snf2-related CREBBP activator protein |
| Chr7 | 127542088 | 127542287 | 200 | Exon | Srcap | Snf2-related CREBBP activator protein |
| Chr7 | 127908187 | 127908531 | 345 | 3' UTR | Bckdk | branched-chain ketoacid dehydrogenase kinase |
| Chr7 | 133719220 | 133720847 | 1628 | Exon | Bccip | BRCA2 and CDKN1A interacting protein |
| Chr7 | 140846223 | 140846721 | 499 | 3' UTR | Scgb1c1 | secretoglobin, family 1C, member 1 |
| Chr7 | 3665904 | 3666146 | 243 | 3' UTR | Tmc4 | transmembrane channel-like gene family 4 |
| Chr7 | 16272161 | 16272655 | 495 | 3' UTR | Inafm1 | InaF motif containing 1 |
| Chr7 | 16916850 | 16917095 | 246 | 3' UTR | Calm3 | calmodulin 3 |
| Chr7 | 27207269 | 27207615 | 347 | 3' UTR | Itpkc | inositol 1,4,5-trisphosphate 3-kinase C |
| Chr7 | 27207714 | 27214511 | 6798 | 3' UTR | Itpkc | inositol 1,4,5-trisphosphate 3-kinase C |
| Chr7 | 27962332 | 27962778 | 447 | 3' UTR | Zfp780b | zinc finger protein 780B |
| Chr7 | 27963672 | 27963920 | 249 | Exon | Zfp780b | zinc finger protein 780B |
| Chr7 | 35276790 | 35287190 | 10401 | Exon | Gpatch1 | G patch domain containing 1 |
| Chr7 | 42661006 | 42661505 | 500 | 3' UTR | Gm5595 | predicted gene 5595 |
| Chr7 | 45031038 | 45034104 | 3067 | Exon | Prr12 | proline-rich 12 |
| Chr7 | 45046053 | 45047897 | 1845 | Exon | Prr12 | proline-rich 12 |
| Chr7 | 45125661 | 45126133 | 473 | 3' UTR | Rpl13a | ribosomal protein L13A |
| Chr7 | 63887450 | 63887700 | 251 | 3' UTR | Klf13 | Kruppel-like factor 13 |
| Chr7 | 79875424 | 79875821 | 398 | 3' UTR | Ap3s2 | adaptor-related protein complex 3, sigma 2 subunit |
| Chr7 | 83882093 | 83883692 | 1600 | 5' UTR | Mesdc1 | mesoderm development candidate 1 |
| Chr7 | 100930211 | 100931011 | 801 | Exon | Arhgef17 | Rho guanine nucleotide exchange factor (GEF) 17 |
| Chr7 | 118489737 | 118491876 | 2140 | 5' UTR | Itpripl2 | inositol 1,4,5-triphosphate receptor interacting protein-like 2 |
| Chr7 | 127015100 | 127017303 | 2204 | 5' UTR | Pagr1a | PAXIP1-associated glutamate-rich protein 1A |
| Chr7 | 127200397 | 127203462 | 3066 | Exon | Tbc1d10b | TBC1 domain family, member 10b |
| Chr7 | 127203820 | 127208221 | 4402 | Exon | Tbc1d10b | TBC1 domain family, member 10b |
| Chr7 | 128697781 | 128698129 | 349 | 3' UTR | Mcmbp | minichromosome maintenance complex-binding protein |
| Chr7 | 132557867 | 132560779 | 2913 | 3' UTR | Oat | ornithine aminotransferase |
| Chr7 | 141638244 | 141638642 | 399 | Exon | Muc6 | mucin 6, gastric |
| Chr7 | 141638742 | 141639091 | 350 | Exon | Muc6 | mucin 6, gastric |
| Chr7 | 142375965 | 142376209 | 245 | 3' UTR | Ctsd | cathepsin D |
| Chr7 | 142491130 | 142492327 | 1198 | Exon | Lsp1 | lymphocyte specific 1 |
| Chr8 | 3621601 | 3623540 | 1940 | 3' UTR | Pcp2 | Purkinje cell protein 2 (L7) |
| Chr8 | 11443203 | 11443627 | 425 | Exon | Col4a2 | collagen, type IV, alpha 2 |
| Chr8 | 71913597 | 71913796 | 200 | Exon | Zfp882 | zinc finger protein 882 |
| Chr8 | 71922925 | 71932632 | 9708 | 5' UTR | Zfp617 | zinc finger protein 617 |
| Chr8 | 72171288 | 72176339 | 5052 | Exon | Rab8a | RAB8A, member RAS oncogene family |
| Chr8 | 83660997 | 83661242 | 246 | Exon | Gipc1 | GIPC PDZ domain-containing family, member 1 |
| Chr8 | 84913433 | 84920493 | 7061 | 5' UTR | Mast1 | microtubule-associated serine/threonine kinase 1 |
| Chr8 | 94172757 | 94173428 | 672 | 5' UTR | Mt2 | metallothionein 2 |
| Chr8 | 94778122 | 94780332 | 2211 | Exon | Cx3cl1 | chemokine (C-X3-C motif) ligand 1 |
| Chr8 | 94780431 | 94780631 | 201 | 3' UTR | Cx3cl1 | chemokine (C-X3-C motif) ligand 1 |
| Chr8 | 95352395 | 95352594 | 200 | 5' UTR | Mmp15 | matrix metallopeptidase 15 |
| Chr8 | 95371076 | 95372655 | 1580 | 3' UTR | Mmp15 | matrix metallopeptidase 15 |
| Chr8 | 105900524 | 105930654 | 30131 | 5' UTR | Pskh1 | protein serine kinase H1 |
| Chr8 | 123095200 | 123097751 | 2552 | 3' UTR | Spg7 | spastic paraplegia 7 homolog (human) |
| Chr8 | 123662939 | 123663831 | 893 | 3' UTR | Rhou | ras homolog family member U |
| Chr8 | 11006002 | 11006894 | 893 | Exon | Irs2 | insulin receptor substrate 2 |
| Chr8 | 23140170 | 23140901 | 732 | Exon | Ank1 | ankyrin 1, erythroid |
| Chr8 | 25767686 | 25768286 | 601 | 3' UTR | Bag4 | BCL2-associated athanogene 4 |
| Chr8 | 70513445 | 70513688 | 244 | 3' UTR | Kxd1 | KxDL motif containing 1 |
| Chr8 | 71456998 | 71458054 | 1057 | 3' UTR | Abhd8 | abhydrolase domain containing 8 |
| Chr8 | 71461618 | 71461767 | 150 | Exon | Abhd8 | abhydrolase domain containing 8 |
| Chr8 | 80705258 | 80709755 | 4498 | Exon | Smarca5 | SWI/SNF-related, matrix-associated, actin-dependent regulator of chromatin, subfamily a, member 5 |
| Chr8 | 85546563 | 85548900 | 2338 | Exon | Dnaja2 | DnaJ heat shock protein family (Hsp40) member A2 |
| Chr8 | 119809363 | 119809561 | 199 | 3' UTR | Cotl1 | coactosin-like 1 (Dictyostelium) |
| Chr8 | 121882383 | 121882877 | 495 | 3' UTR | Slc7a5 | solute carrier family 7 (cationic amino acid transporter, y+ system), member 5 |
| Chr8 | 122574733 | 122575413 | 681 | 3' UTR | Aprt | adenine phosphoribosyl transferase |
| Chr8 | 123841091 | 123841289 | 199 | 3' UTR | Ccsap | centriole-, cilia-, and spindle-associated protein |
| Chr8 | 124556736 | 124556935 | 200 | 3' UTR | Agt | angiotensinogen (serpin peptidase inhibitor, clade A, member 8) |
| Chr8 | 124895319 | 124896959 | 1641 | 3' UTR | Exoc8 | exocyst complex component 8 |
| Chr9 | 21019076 | 21025838 | 6763 | Exon | Icam1 | intercellular adhesion molecule 1 |
| Chr9 | 21593445 | 21594187 | 743 | 3' UTR | 1810026J23Rik | RIKEN cDNA 1810026J23 gene |
| Chr9 | 22157763 | 22158306 | 544 | 5' UTR | Pigyl | phosphatidylinositol glycan anchor biosynthesis, class Y-like |
| Chr9 | 43804435 | 43804633 | 199 | 3' UTR | Nectin1 | nectin cell adhesion molecule 1 |
| Chr9 | 51849082 | 51850673 | 1592 | 3' UTR | Arhgap20 | Rho GTPase activating protein 20 |
| Chr9 | 56937550 | 56938348 | 799 | 3' UTR | Imp3 | IMP3, U3 small nucleolar ribonucleoprotein |
| Chr9 | 58488702 | 58499643 | 10942 | 5' UTR | 6030419C18Rik | RIKEN cDNA 6030419C18 gene |
| Chr9 | 62781955 | 62783480 | 1526 | 3' UTR | Itga11 | integrin alpha 11 |
| Chr9 | 65452058 | 65452606 | 549 | 3' UTR | Mtfmt | mitochondrial methionyl-tRNA formyltransferase |
| Chr9 | 89199416 | 89207647 | 8232 | Exon | Bcl2a1b | B cell leukemia/lymphoma 2-related protein A1b |
| Chr9 | 107587866 | 107590540 | 2675 | 5' UTR | Ifrd2 | interferon-related developmental regulator 2 |
| Chr9 | 108048386 | 108048732 | 347 | 3' UTR | Ip6k1 | inositol hexaphosphate kinase 1 |
| Chr9 | 110767909 | 110768058 | 150 | Exon | Myl3 | myosin, light polypeptide 3 |
| Chr9 | 111468705 | 111469154 | 450 | Exon | Dclk3 | doublecortin-like kinase 3 |
| Chr9 | 114097889 | 114098385 | 497 | 3' UTR | Susd5 | sushi domain containing 5 |
| Chr9 | 15005884 | 15008014 | 2131 | 3' UTR | Panx1 | pannexin 1 |
| Chr9 | 21768555 | 21768704 | 150 | 3' UTR | Kank2 | KN motif and ankyrin repeat domains 2 |
| Chr9 | 21794576 | 21795170 | 595 | Exon | Kank2 | KN motif and ankyrin repeat domains 2 |
| Chr9 | 21795269 | 21798496 | 3228 | 5' UTR | Kank2 | KN motif and ankyrin repeat domains 2 |
| Chr9 | 43239617 | 43239766 | 150 | 5' UTR | Oaf | out at first homolog |
| Chr9 | 44111274 | 44112755 | 1482 | 3' UTR | Rnf26 | ring finger protein 26 |
| Chr9 | 44247362 | 44249022 | 1661 | 3' UTR | Pdzd3 | PDZ domain containing 3 |
| Chr9 | 100458313 | 100466231 | 7919 | 3' UTR | Il20rb | interleukin 20 receptor beta |
| Chr9 | 108575262 | 108578570 | 3309 | Exon | Wdr6 | WD repeat domain 6 |
| Chr9 | 119927762 | 119928251 | 490 | 3' UTR | Gorasp1 | golgi reassembly stacking protein 1 |
| ChrX | 9265803 | 9266250 | 448 | 3' UTR | Lancl3 | LanC lantibiotic synthetase component C-like 3 (bacterial) |
| ChrX | 37136512 | 37139712 | 3201 | Exon | Nkap | NFKB activating protein |
| ChrX | 74311621 | 74311818 | 198 | 3' UTR | Gdi1 | guanosine diphosphate (GDP) dissociation inhibitor 1 |
| ChrX | 99136160 | 99136802 | 643 | 5' UTR | Efnb1 | ephrin B1 |
| ChrX | 99145376 | 99147837 | 2462 | 3' UTR | Efnb1 | ephrin B1 |
| ChrX | 102119560 | 102127578 | 8019 | Exon | Pin4 | protein (peptidyl-prolyl cis/trans isomerase) NIMA-interacting, 4 (parvulin) |
| ChrX | 105764676 | 105774489 | 9814 | 3' UTR | Fgf16 | fibroblast growth factor 16 |
| ChrX | 135745742 | 135746287 | 546 | Exon | Armcx5 | armadillo repeat containing, X-linked 5 |
| ChrX | 136245177 | 136246509 | 1333 | 5' UTR | Wbp5 | WW domain-binding protein 5 |
| ChrX | 153498679 | 153498878 | 200 | Exon | Ubqln2 | ubiquilin 2 |
| ChrX | 153498978 | 153499574 | 597 | Exon | Ubqln2 | ubiquilin 2 |
| ChrX | 153499673 | 153500270 | 598 | Exon | Ubqln2 | ubiquilin 2 |
| ChrX | 94540460 | 94541927 | 1468 | 5' UTR | Maged1 | melanoma antigen, family D, 1 |
| ChrX | 109005026 | 109013331 | 8306 | 5' UTR | Hmgn5 | high-mobility group nucleosome-binding domain 5 |
| ChrX | 134972408 | 134973938 | 1531 | 3' UTR | Zmat1 | zinc finger, matrin type 1 |

**Supplementary Table S2. Echocardiographic parameters**

|  | **IVS;d** | **p value** | **LVID;d** | **p value** | **LVPW;d** | **p value** | **IVS;s** | **p value** | **LVID;s** | **p value** | **LVPW;s** | **p value** | **EF (%)** | **p value** | **FS (%)** | **p value** | **HR** | **p value** |
| --- | --- | --- | --- | --- | --- | --- | --- | --- | --- | --- | --- | --- | --- | --- | --- | --- | --- | --- |
| 2wk sham | 1.070±0.047 |  | 3.685±0.079 |  | 0.836±0.079 |  | 1.536±0.044 |  | 2.518±0.099 |  | 1.177±0.084 |  | 65.361±3.206 |  | 35.559±2.557 |  | 506.833±20.771 |  |
| 2wk TAC | 1.200±0.070 | 0.162 | 3.625±0.091 | 0.636 | 1.083±0.095 | 0.076 | 1.633±0.052 | 0.192 | 2.633±0.126 | 0.500 | 1.358±0.088 | 0.168 | 56.920±2.857 | 0.074 | 29.417±1.867 | 0.073 | 530.286±30.665 | 0.554 |
| 4wk sham | 1.050±0.086 |  | 3.520±0.104 |  | 0.836±0.042 |  | 1.459±0.066 |  | 2.411±0.101 |  | 1.128±0.049 |  | 66.604±1.968 |  | 36.191±1.527 |  | 481.167±28.726 |  |
| 4wk TAC | 1.293±0.109 | 0.112 | 3.617±0.167 | 0.632 | 1.215±0.083 | 0.002 | 1.760±0.083 | 0.017 | 2.567±0.189 | 0.483 | 1.507±0.109 | 0.010 | 50.067±1.989 | <0.001 | 25.072±1.192 | <0.001 | 512.833±39.899 | 0.534 |
| 8wk sham | 0.992±0.062 |  | 3.743±0.059 |  | 0.846±0.036 |  | 1.381±0.063 |  | 2.664±0.091 |  | 1.120±0.093 |  | 61.279±3.310 |  | 32.616±2.434 |  | 467.000±25.529 |  |
| 8wk TAC | 1.157±0.118 | 0.244 | 4.025±0.335 | 0.426 | 1.079±0.069 | 0.013 | 1.624±0.129 | 0.122 | 3.024±0.412 | 0.414 | 1.467±0.119 | 0.044 | 39.631±4.817 | 0.004 | 19.498±2.549 | 0.004 | 537.500±32.185 | 0.117 |
|  |  |  |  |  |  |  |  |  |  |  |  |  |  |  |  |  |  |  |
| 2wk sham ctrl | 1.000±0.072 |  | 3.805±0.066 |  | 0.795±0.042 |  | 1.430±0.117 |  | 2.640±0.158 |  | 1.130±0.053 |  | 62.106±3.480 |  | 33.371±2.645 |  | 523.143±15.931 |  |
| 2wk FTO-KD | 1.053±0.084 | 0.965 | 4.041±0.066 | 0.311 | 0.780±0.037 | 0.997 | 1.410±0.101 | 0.999 | 3.004±0.055 | 0.096 | 1.018±0.041 | 0.302 | 53.389±1.410 | 0.111 | 27.233±0.924 | .073 | 467.364±16.653 | 0.399 |
| 2wk TAC ctrl | 1.360±0.039 |  | 3.735±0.083 |  | 1.230±0.091 |  | 1.715±0.059 |  | 2.925±0.089 |  | 1.470±0.049 |  | 50.694±4.059 |  | 26.394±2.316 |  | 477.857±41.833 |  |
| 2wk TAC FTO-KD | 1.435±0.108 | 0.932 | 3.795±0.168 | 0.977 | 1.210±0.065 | 0.996 | 1.840±0.129 | 0.865 | 2.970±0.154 | 0.993 | 1.445±0.039 | 0.984 | 50.117±2.341 | 0.999 | 25.121±1.382 | 0.963 | 503.143±27.692 | 0.914 |
| 4wk sham ctrl | 1.005±0.078 |  | 3.650±0.150 |  | 0.935±0.106 |  | 1.500±0.091 |  | 2.565±0.183 |  | 1.230±0.070 |  | 66.136±4.071 |  | 36.450±3.020 |  | 526.429±21.465 |  |
| 4wk FTO-KD | 1.060±0.103 | 0.968 | 3.891±0.113 | 0.488 | 0.770±0.024 | 0.148 | 1.527±0.079 | 0.995 | 2.771±0.104 | 0.646 | 1.066±0.048 | 0.147 | 58.551±1.968 | 0.238 | 30.629±1.327 | 0.137 | 482.455±13.521 | 0.489 |
| 4wk TAC ctrl | 1.280±0.065 |  | 3.877±0.059 |  | 1.000±0.160 |  | 1.686±0.096 |  | 3.146±0.070 |  | 1.256±0.067 |  | 43.403±3.066 |  | 21.285±1.717 |  | 533.667±31.573 |  |
| 4wk TAC FTO-KD | 1.278±0.041 | 0.963 | 4.171±0.115 | 0.460 | 1.299±0.043 | 0.013 | 1.622±0.050 | 0.963 | 3.640±0.150 | 0.110 | 1.448±0.028 | 0.161 | 32.786±3.178 | 0.131 | 15.443±1.638 | 0.257 | 569.500±34.060 | 0.762 |
| 8wk sham ctrl | 0.965±0.044 |  | 3.785±0.068 |  | 0.750±0.018 |  | 1.605±0.068 |  | 2.445±0.063 |  | 1.070±0.042 |  | 69.764±2.200 |  | 38.955±1.842 |  | 551.286±18.454 |  |
| 8wk FTO-KD | 0.938±0.061 | 0.988 | 4.025±0.084 | 0.685 | 0.760±0.025 | 0.999 | 1.404±0.054 | 0.393 | 2.720±0.102 | 0.666 | 1.113±0.041 | 0.931 | 63.276±2.561 | 0.209 | 34.268±1.906 | 0.196 | 521.400±13.352 | 0.717 |
| 8wk TAC ctrl | 1.107±0.057 |  | 4.579±0.062 |  | 0.911±0.018 |  | 1.433±0.168 |  | 3.803±0.082 |  | 1.233±0.006 |  | 38.263±1.054 |  | 18.465±0.579 |  | 578.833±28.097 |  |
| 8wk TAC FTO-KD | 1.313±0.081 | 0.178 | 5.343±0.358 | 0.027 | 1.174±0.117 | 0.016 | 1.604±0.101 | 0.653 | 4.848±0.392 | 0.005 | 1.457±0.094 | 0.049 | 24.660±2.821 | 0.007 | 11.506±1.378 | 0.007 | 516.500±30.561 | 0.260 |
|  |  |  |  |  |  |  |  |  |  |  |  |  |  |  |  |  |  |  |
| 2wk sham ctrl | 1.036±0.061 |  | 3.710±0.105 |  | 0.705±0.037 |  | 1.436±0.065 |  | 2.668±0.085 |  | 1.015±0.065 |  | 62.502±1.066 |  | 32.449±0.485 |  | 509.556±20.312 |  |
| 2wk aavFTO | 1.030±0.100 | 0.999 | 3.612±0.111 | 0.895 | 0.805±0.023 | 0.447 | 1.407±0.113 | 0.993 | 2.578±0.153 | 0.945 | 0.985±0.024 | 0.988 | 64.280±1.968 | 0.909 | 34.105±1.509 | 0.836 | 490.000±20.152 | 0.901 |
| 2wk TAC ctrl | 1.070±0.086 |  | 3.617±0.099 |  | 0.934±0.064 |  | 1.575±0.060 |  | 2.586±0.103 |  | 1.235±0.096 |  | 57.609±1.893 |  | 29.808±1.230 |  | 507.000±16.371 |  |
| 2wk TAC aavFTO | 1.175±0.035 | 0.769 | 3.532±0.088 | 0.946 | 1.032±0.066 | 0.544 | 1.645±0.083 | 0.941 | 2.507±0.136 | 0.970 | 1.357±0.077 | 0.642 | 64.306±2.899 | 0.133 | 34.754±2.324 | 0.133 | 506.000±24.294 | 0.999 |
| 4wk sham ctrl | 1.071±0.045 |  | 3.643±0.058 |  | 0.846±0.033 |  | 1.631±0.060 |  | 2.348±0.088 |  | 1.200±0.067 |  | 68.015±1.473 |  | 37.378±1.157 |  | 524.667±17.966 |  |
| 4wk aavFTO | 1.015±0.057 | 0.917 | 3.668±0.080 | 0.994 | 0.953±0.085 | 0.688 | 1.490±0.027 | 0.479 | 2.517±0.056 | 0.481 | 1.277±0.080 | 0.941 | 64.891±1.647 | 0.640 | 34.998±1.229 | 0.556 | 494.857±16.826 | 0.884 |
| 4wk TAC ctrl | 1.309±0.077 |  | 4.069±0.056 |  | 1.200±0.114 |  | 1.703±0.121 |  | 3.032±0.034 |  | 1.422±0.155 |  | 46.154±2.762 |  | 22.818±1.640 |  | 510.667±45.681 |  |
| 4wk TAC aavFTO | 1.218±0.082 | 0.779 | 3.760±0.104 | 0.055 | 0.952±0.058 | 0.117 | 1.572±0.068 | 0.613 | 2.778±0.121 | 0.227 | 1.395±0.106 | 0.998 | 55.096±2.083 | 0.024 | 28.241±1.350 | 0.052 | 488.857±39.634 | 0.962 |
| 8wk sham ctrl | 1.129±0.086 |  | 3.860±0.120 |  | 0.782±0.070 |  | 1.625±0.092 |  | 2.585±0.178 |  | 1.203±0.102 |  | 63.577±2.983 |  | 34.488±2.339 |  | 475.333±22.850 |  |
| 8wk aavFTO | 0.963±0.048 | 0.495 | 3.928±0.085 | 0.974 | 0.856±0.067 | 0.795 | 1.458±0.077 | 0.641 | 2.552±0.143 | 0.999 | 1.212±0.102 | 0.999 | 67.505±2.781 | 0.741 | 37.392±2.278 | 0.718 | 512.143±15.603 | 0.605 |
| 8wk TAC ctrl | 1.126±0.110 |  | 4.870±0.132 |  | 1.241±0.054 |  | 1.388±0.172 |  | 4.272±0.215 |  | 1.221±0.065 |  | 32.650±3.575 |  | 15.599±1.868 |  | 538.000±24.310 |  |
| 8wk TAC aavFTO | 1.170±0.089 | 0.986 | 4.263±0.130 | 0.011 | 0.975±0.025 | 0.032 | 1.543±0.072 | 0.753 | 3.392±0.136 | 0.10 | 1.300±0.061 | 0.936 | 47.545±1.662 | 0.009 | 25.113±0.793 | 0.021 | 505.143±22.099 | 0.747 |

Supplemental Table S2. All echocardiographic parameters collected (mean ± SEM) for all experiments. IVS;d = interventricular septum thickness; diastole. LVID;d = left ventricular internal dimension; diastole. LVPW;d = left ventricular posterior wall thickness; diastole. IVS;s = interventricular septum thickness; systole. LVID;s = left ventricular internal dimension; systole. LVPW;s = left ventricular posterior wall thickness; systole. EF = ejection fraction. FS = fractional shortening. HR = heart rate.
